# Challenges and opportunities in predicting coastal total water levels from empirical runup models: Insights from major storms in Southeast Australia

**DOI:** 10.1017/cft.2026.10043

**Published:** 2026-06-03

**Authors:** Raimundo Ibaceta, David Hanslow, Michael Hughes, Bradley Morris, Michael A. Kinsela, Timothy Ingleton, Neil Dospotz, Stephen Holtznagel, Cristina Viola, Danial Khojasteh

**Affiliations:** 1 NSW Department of Climate Change, Energy, the Environment and Water, Australia; 2 School of Earth Atmospheric and Life Sciences, University of Wollongong, North Wollongong, NSW, Australia; 3 Earth Sciences, School of Science, University of Newcastle, Callaghan, NSW, Australia

**Keywords:** inundation, runup, beach slope, LiDAR, CoastSat

## Abstract

Total water levels (*TWL*) – the combination of wave-runup, tides, surges and in the future, sea-level rise – stand as a critical variable contributing to coastal inundation during storms. Quantifying the contribution of wave runup to extreme *TWL* is essential for accurate assessments of coastal inundation risk at wave-dominated coastlines. Despite decades of research, a fundamental challenge for coastal practitioners is selecting from numerous runup models, often constrained by the unavailable site-specific beach slope data. This study presents a novel dataset of *TWL* observations inferred from marine debris deposits surveyed immediately after storms in New South Wales, Australia. Using this dataset, this work assesses the accuracy of simplified *TWL* models integrating still water levels, local nearshore wave data, various runup formulae and different beach slope proxies derived from remote sensing technologies. Models utilising historical beach slopes from LiDAR outperformed the models using satellite-derived slopes. Findings suggest that runup formulae calibrated during small-to-moderate wave conditions can be extrapolated to extreme storms when applied to similar sites, emphasising the preference for site-specific formulae over one-model-fits-all approaches. Challenges and opportunities of modelling *TWL* for coastal inundation assessments are discussed, highlighting the need to incorporate beach slope uncertainties to fully assess inundation impacts as sea-levels rise.

## Impact statement

During coastal storms, waves and storm tides push water inland, increasing the risk to homes, roads, dunes and beaches. Knowing how high the water will reach is therefore essential for preparing for storms and planning for future sea-level rise. However, this is hard to predict, especially because there are many existing methods that can work well for some conditions but not so good elsewhere. This study improves that picture by using evidence left behind after storms, such as lines of marine debris, to estimate how far the water reached along beaches in New South Wales, Australia. These observations were then used to test different ways of predicting storm water levels. The study shows that some simple prediction methods work better than others. In particular, models based on detailed airborne laser measurements of beach slope were more accurate than those using newer satellite-based estimates. It also found that a formula developed specifically for beaches in southeast Australia performed well even during very large storms. The wider importance of this research is that it gives coastal planners and emergency managers better guidance on how to estimate storm flooding at sandy beaches. It also shows that storm impacts can be studied using practical field evidence collected after an event, even when direct measurements during the storm are difficult. As sea levels rise and coastal populations grow, better estimates of how far storm water can reach will help communities make smarter decisions about development, protection and climate adaptation.

## Highlights

- Large regional dataset of surveyed marine debris lines following storms in south-eastern Australia shows regional and intra-beach variability in extreme total water levels (*TWLs*).
- Extreme *TWL* is modelled using tidal gauges, local nearshore wave data, several empirical runup formulae and beach slope specifications from LiDAR and satellites.
- Large variability in model accuracy occurs across different runup formulae and beach slope specifications.
- Beach slope uncertainty is leveraged to account for modelling errors from deterministic slopes
- Errors from present-day *TWL* modelling can exceed the magnitudes and uncertainties of projected sea level rise.

## Introduction

Coastal storms can inflict widespread damage on infrastructure and the environment, posing significant threats to coastal communities and economies. Among the primary storm hazards is coastal inundation caused by elevated water levels, which can lead to flooding in low-lying areas, damage to adjacent beach infrastructure and, in extreme cases, loss of life. With increasing anthropogenic pressures on coastal areas (Neumann et al., 2015; Jones and O’Neill, 2016), sea level rise (SLR) (Kulp and Strauss, 2019; Hague et al., 2020; Khojasteh et al., 2023) and uncertainties regarding future trends in extreme storm events (Mentaschi et al., 2017; Reguero et al., 2019; Meucci et al., 2020; Morim et al., 2021; Casas-Prat et al., 2024), the risk from elevated water levels is likely to exacerbate (Vitousek et al., 2017; Taherkhani et al., 2020), threatening local infrastructure along developed coastlines globally (Vousdoukas et al., 2018; Barnard et al., 2019a; Almar et al., 2021; Casas-Prat et al., 2024). Enhancing our knowledge and predictive capabilities of coastal inundation is therefore crucial to inform future studies supporting coastal management, disaster risk reduction and adaptation strategies (Toimil et al., 2023, 2020).

**Figure 1.**
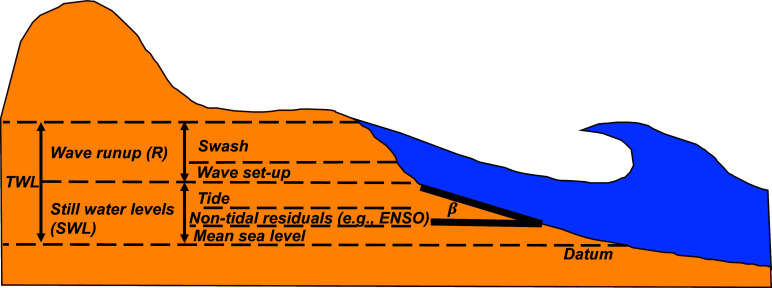
A conceptual schematic highlighting the contributing factors to TWLs, including runup (swash and set-up) and still water levels (MSL, tides and non-tidal residuals), at sandy coastlines under present conditions. Figure 1. long description.

Quantifying extreme coastal water levels during storms is the baseline for coastal inundation hazard assessments. Coastal practitioners and planners rely on extreme water level predictions to identify low-lying areas vulnerable to flooding (Almar et al., 2021; Benito et al., 2024; Chtioui et al., 2024), plan future coastal developments (Mather et al., 2011), and estimate berm and dune erosion (Beuzen et al., 2019; Oliver et al., 2024; Palmsten and Holman, 2012), among other applications. Along global wave-dominated sandy coastlines characterised by narrow continental shelves (Nyberg and Howell, 2016), coastal water levels are primarily influenced by wave runup and to a lesser extent by astronomical tides and non-tidal residuals (Leaman et al., 2021). The most widely used proxy that aggregates these processes is the *total water level* (*TWL*) variable. *TWL* is defined as the sum of (dynamic) still water levels (*SWL*) comprising tides and non-tidal residuals, and wave runup (*R*), resulting in *TWL = SWL + R* (e.g., Almar et al., 2021; Conlin et al., 2023; Zornoza-Aguado et al., 2024), as illustrated in Figure 1.

*SWL* include variations from mean sea level (MSL), astronomical tides (e.g., the spring-neap cycle) and non-tidal residuals (e.g., storm surge, interannual climatic effects) (Viola et al., 2021), typically monitored at water level stations. Alternatively, coastal practitioners use global tide models (e.g., Carrère et al., 2015; Lyard et al., 2021) and surge models (Muis et al., 2023) to estimate *SWL* at ungauged locations. Wave runup (*R*) levels – the vertical excursion of waves at the shoreline – include a time-averaged component (wave set-up) and an oscillating component of the water line (swash), commonly measured with video techniques (Holman and Stanley, 2007) or laser scanning (Phillips et al., 2019; Kim et al., 2023).

Measuring wave runup during storms is a challenging task due to the adverse weather conditions accompanying these extreme events. Video monitoring systems struggle to detect swash motions effectively under high wind and rainy conditions (Holman and Haller, 2013; Almeida et al., 2015; Kim et al., 2023). In contrast, laser scanning instruments (Blenkinsopp et al., 2010; Phillips et al., 2019) overcome weather/climate-related issues as they continuously measure instantaneous water levels (Almeida et al., 2015; Beuzen et al., 2019; Kim et al., 2023). However, this technology measures wave transformation only at individual cross-shore transects (Martins et al., 2017), limiting its applicability to study extreme *TWL* at large scales. An alternative approach to measure extreme *TWL* at larger spatial scales involves surveying the position (along the beach) of the maximum elevation reached by the swash lens after a storm. Similar to other areas of coastal research (e.g., tsunami science, see for instance, Fritz et al., 2011), the maximum swash limit can be identified from landward marine debris deposits and/or wet/dry transitions that correspond to peak *TWL* during storms (Cariolet and Suanez, 2013; Suanez et al., 2015). The widespread availability of real-time kinematic GPS technology (Harley et al., 2011a) enhances this method’s application for measuring extreme *TWL* across large scales (hundreds of km), valuable for coastal risk assessments and benchmarking of existing *TWL* modelling approaches (e.g., Atkinson et al., 2017; Gomes da Silva et al., 2020).

The widespread availability of runup datasets across the Americas, Australasia and Europe has led to the development of many empirical formulae for predicting wave runup statistics (e.g., Ruggiero et al., 2001; Stockdon et al., 2006; Atkinson et al., 2017; Gomes da Silva et al., 2020). Most of these formulae estimate *R_2%_* (i.e., the elevation exceeded by 2% of waves) based on offshore wave properties (*H_o_* and *L_o_*, offshore significant wave height and wave length, respectively) and a single value representative of the beach face slope (*β*) (Figure 1). Originally derived for planar beaches in the laboratory (Hunt, 1959), the ‘Hunt’ scaling relationship (*R* $\propto \tan\left(\beta \right)\sqrt{H_{o}L_{o}}$ ) is typically fitted to local data characterising site-specific runup dynamics (Vousdoukas et al., 2012; Gomes da Silva et al., 2020; Portch et al., 2023). Compared to more complex process-based models of coastal inundation (e.g., Xbeach, Roelvink et al., 2009), empirical models are widely used by coastal practitioners as they can be readily applied from local to large regional scales and extrapolated to different wave and beach slope characteristics (Leaman et al., 2021; Shope et al., 2022; Stockdon et al., 2023). Stockdon et al. (2006) presented a generalised *R_2%_* model derived from observations of 10 field experiments across a wide range of beach characteristics (*β* ranging from 0.01 to 0.11) and low to moderate wave energy (*H_o_* ranging from 0.7 m to 2.5 m). This ‘one-model-fits-all’ formula has arguably become a standard in coastal inundation risk analysis (e.g., Vitousek et al., 2017; Almar et al., 2021; Stockdon et al., 2023). In comparing their 2006 formula with a more complex process-based numerical model (Xbeach), Stockdon et al. (2014) concluded adjustments were needed for this formula to perform well under storm conditions (*H_o_* ~ 3 m), as observed during the SandyDuck field experiment (Stockdon et al., 2011). Atkinson et al. (2017) tested 9 existing runup models against a blind dataset from 11 beaches in south-eastern (SE) Australia. They found that no single formula performed consistently across all 11 beaches and proposed a ‘model-of-models’ approach that fitted a Hunt-type expression to the predictions from all nine earlier models. This model-of-models was the most accurate at predicting *R_2%_* jointly with previous site-specific formulae by Vousdoukas et al. (2012) and Holman (1986), although it was not tested under storm conditions (Atkinson et al., 2017). Recent studies have compiled larger datasets that include storm events to train machine learning models for predicting *R_2%_* (e.g., genetic programming, Gaussian models, XGBoost, Beuzen et al. 2019; Power et al., 2019, Kim and Lee, 2024). While these approaches perform well within their training data, their accuracy outside the training/learning range may lead to large model errors (Beuzen and Splinter, 2020; Kim and Lee, 2024).

**Figure 2.**
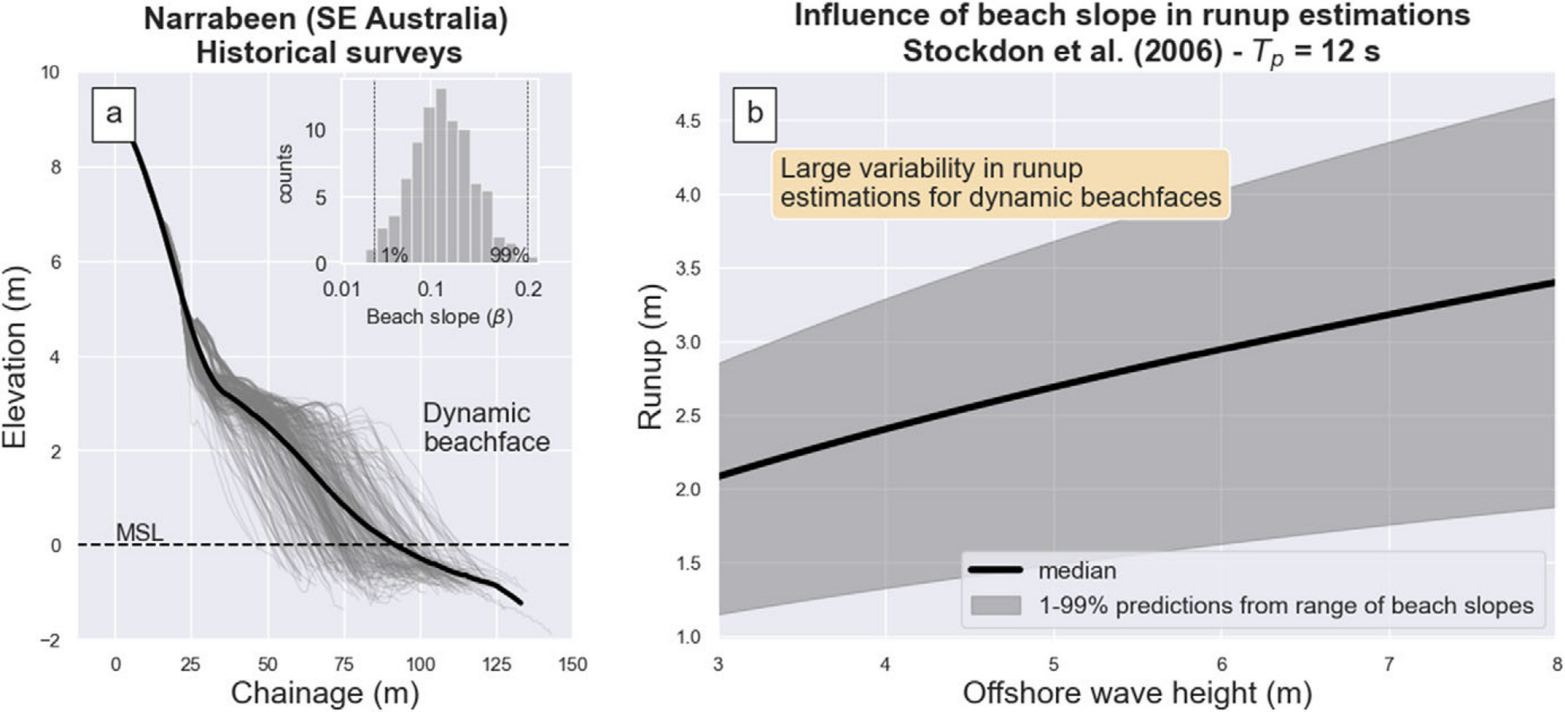
This example shows the challenges of estimating runup at dynamic beaches during storms. (a) Long-term (1976–2021) cross-shore transects at Narrabeen Beach (SE Australia) reveal large variability in beach slopes at this dynamic, intermediate beach (see inset – beach slopes were calculated between the MSL and 2 m, a proxy for the berm elevation). (b) Large variability in runup estimations during storms (Ho – 3 to 8 m, Tp = 12 s) resulting from highly variable beach slopes. Runup magnitudes were estimated using the widely adopted model of Stockdon et al. (2006). Figure 2. long description.

Despite the development of numerous site-specific and generalised runup formulae over the past decades, coastal practitioners face the daunting task of selecting the most suitable model from over 30+ options (Supplementary Appendix A in Gomes da Silva et al., 2020). Moreover, adequate specification of the beach slope magnitude (*β*) is key in the accuracy of coastal inundation risk studies (Aucan et al., 2019; O’Grady et al., 2019) and its misspecification has been recently reported as the greatest contributor to runup modelling errors (Kim and Lee, 2024). Nonetheless, a universal definition of *β* is still absent in the literature. Kim et al. (2023) used laser scanning technology to test different beach slope proxies for estimating storm runup at the Duck Field Research Facility (*H_o_* ~ 4 m). They found that pre-storm beach slope measurements can provide accurate runup estimates when compared to more complex models that consider morphological changes during storms. Unfortunately, pre-storm morphological data are often unavailable elsewhere (Simmons et al., 2019; Matheen et al., 2021), forcing practitioners to rely on *β* values from any available (often isolated) surveys at their study sites. As illustrated in Figure 2, selecting appropriate beach slope magnitudes is essential, particularly for highly dynamic coastlines where the natural temporal variability in beach face morphology can lead to significant differences in runup estimates, often varying by several metres.

The advent of remote sensing technology is revolutionising the availability of beach morphological data across extensive spatial and temporal scales (Splinter et al., 2018; Vitousek et al., 2023). Episodic Airborne LiDAR flights provide topographic data at different epochs, enabling the estimation of historical beach slopes and their variability over hundreds of kilometres of coastline (Saye et al., 2005; Stockdon et al., 2009; Harley, 2017). In addition, recent advances in satellite remote sensing technology now offer estimates of long-term intertidal beach slopes from local to continental scales (Vos et al., 2023, 2020, 2019). These modern monitoring techniques call for a timely evaluation of these data sources to enhance modelling of extreme *TWL.* Such improvements are crucial for developing robust methodologies to support coastal practitioners in managing coastal flooding risks, particularly in the context of accelerating SLR.

This study utilises a novel and extensive regional dataset of extreme *TWL* observations collected during four storms events in New South Wales (NSW), SE Australia. These observations are sourced from regional surveys of marine debris lines representing extreme *TWL.* Using this detailed dataset, this work primarily addresses the question: *How do observed still water levels, different runup formulae and new beach slope datasets perform in predicting TWL during storms?*

After assessing the challenges associated with model and data selection, this study then explores the potential to leverage the spatio-temporal variability in beach slopes to predict a wider range of plausible *TWL* observations. The challenges in forward-forecasting extreme *TWL* under rising sea levels are also discussed.

Following a description of the *TWL* dataset, modelling approach and input data in Sections “Data and methods,” “Results” presents the regional scale observations and modelling results, which are discussed in the context of future coastal risk assessments in Section “Discussion.” Concluding remarks are then presented in Section “Conclusions.”

## Data and methods

### Study area and extreme *TWL* dataset

#### Study area: NSW – SE Australia

**Figure 3.**
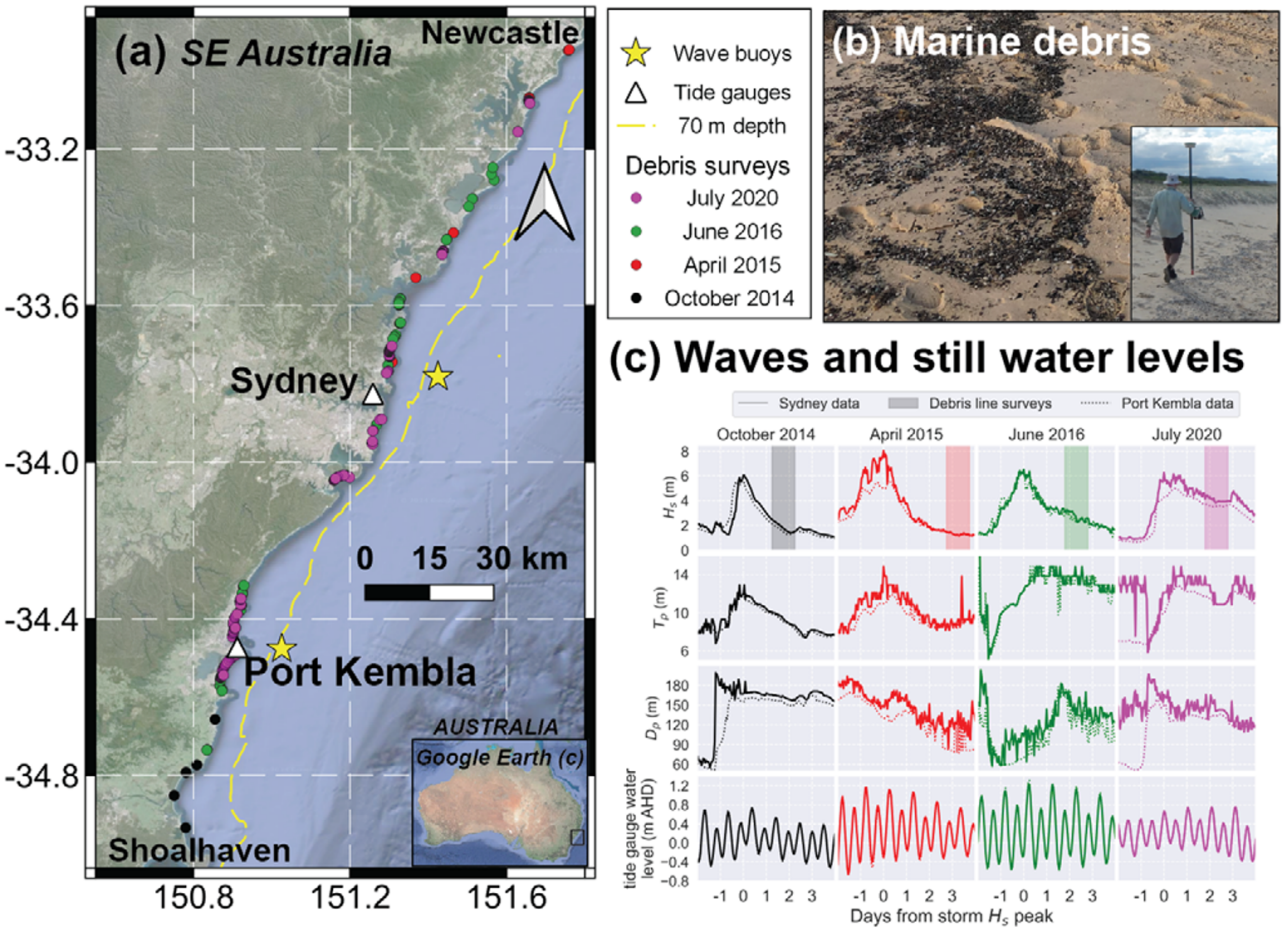
(a) Map of study area in SE Australia, showing the geographical distribution of marine debris surveys conducted after four storm events in October 2014, April 2015, June 2016 and July 2020. Marine debris surveys covered 37 beaches at non-truncated locations, avoiding marine debris deposits at the toe of dune scarps and seawalls. (b) Example of marine debris line and RTK-GNSS monitoring. (c) Time series of offshore wave climate and SWL forcing during all four storm events. Top to bottom panels display offshore wave properties (significant wave height, peak wave period and peak direction) and still water levels obtained from offshore wave buoys (yellow asterisks in panel a) and tide gauges (white triangles in panel a), respectively. Time series are presented for buoys and gauges located in Sydney (solid lines) and Port Kembla (dashed lines). Figure 3. long description.

The study area encompasses ~330 km of coastline in NSW, SE Australia, bounded by Merewether Beach in Newcastle in the north and Shoalhaven Heads in the south (Figure 3a). The region is wave dominated (Short, 2007; Leaman et al., 2021), characterised by energetic swells, microtides and limited surge influence due to the region’s steep and narrow continental shelf (McInnes and Hubbert, 2001). The offshore wave climate is of moderate to high energy (average *H_o_* = 1.6 m and *T_p_* = 9.8 s) and dominated by long-period swell waves from the SSE direction generated in the southern Tasman Sea (Short and Trenaman, 1992). These modal conditions are interrupted approximately 5% of the time by storm waves that exceed a significant wave height threshold of 3 m (Harley et al., 2010; Harley, 2017; Lobeto et al., 2024), with peaks reaching over 8 m during extreme events (Lord and Kulmar, 2000). These storm waves originate from various synoptic patterns, including east coast lows that develop adjacent to the coastline (Dowdy et al., 2019), mid-latitude cyclones from the south and occasionally, tropical cyclones from the northeast (Short and Trenaman, 1992). The region experiences a micro-tidal, semi-diurnal tide with a mean spring tide range of 1.3 m (Beuzen et al., 2019). Non-tidal contributions to SWL vary within ±0.4 m, influenced by surges, coastal trapped waves and multi-year climatic fluctuations (Viola et al., 2024, 2021).

Embayed beaches are prevalent in this region with an average length of 1.3 km and E-ESE orientation (Short, 2007; Fellowes et al., 2019) (Figure 3a). The presence of headlands at the beaches’ southern ends and prevalent SSE swells result in increasing wave energy gradients from south to north along these embayments (Harley et al., 2011b; Fellowes et al., 2022; Ibaceta et al., 2023; Ibaceta and Harley, 2024). Intermediate beach morphologies are common in this region (Wright and Short, 1984; Wright et al., 1985) and typically vary from low-energy and steep sections at southern ends (low-tide terrace) to longshore bar trough, single-bar rhythmic bar-beach and transverse bar-rip states at more exposed central and northern beach sections (Doyle et al., 2024). The regional average intertidal beach slope is approximately 0.06 and typically varies along individual beaches due to changes in wave energy, beach state and grain size (Vos et al., 2022). Intermittently Closed and Open Lake or Lagoons are also prevalent in the region (Khojasteh et al., 2025).

#### Observations of extreme *TWL*

Observations of extreme *TWL* comprise four major meteorological events (referred to for clarity as ‘storms’) in October 2014, April 2015 (e.g., Harley et al., 2016), June 2016 (e.g., Harley, 2017; Mortlock et al., 2017) and July 2020. Hourly offshore wave data were recorded by directional wave buoys located off Sydney and Port Kembla in approximately ~80 m water depth (Figure 3a, yellow asterisks). During these storms, the offshore significant wave height peaked between *H_o_* = 6.0 m and *H_o_* = 8.1 m (Figure 3c, upper panels), corresponding to annual recurrence intervals of 1–20 years (Mortlock et al., 2017). Hourly *SWLs* were measured by nearby tide gauges (Figure 3a, triangles).

Extreme *TWL* measurements were collected from 40 sandy embayed beaches, with 7–28 beaches surveyed per storm (solid circles in Figure 3a). Similar to Cariolet and Suanez (2013) and Suanez et al. (2015), the methodology consisted of tracking the most landward limit between wet and dry sand, which can be easily identified by marine debris deposits immediately after the storm (Figure 3b). Measurements were conducted every ~3 m alongshore the day following the peak in *H_o_* (Figure 3c, shaded blocks) using RTK-GPS and referenced to the local datum (GDA94 projection and vertical Australian Height Datum [AHD]). On this wave-dominated coastline, these measurements correspond to the maximum *TWL* during the storm (*TWL* = *(SWL + R_max_)_max_*), modelled from maximum hourly values of *SWL* and maximum run up *R_max_* for given wave conditions during the storm. This assumes that the debris lines were deposited not at the maximum *SWL* or at the maximum runup (*R_max_*) individually, but at the maximum combined elevation of both during the storm.

To ensure the validity of existing runup formulae, measurements were taken at non-truncated locations, avoiding marine debris deposits at the toe of dune scarps and seawalls. Over 7,000 raw *TWL* measurements were recorded, typically extending across hundreds of metres of beach.

#### Transect dataset and marine debris observations pre-processing

A pre-processing step was applied to raw marine debris observations prior to further analyses and comparison with *TWL* predictions. The SE Australian sandy coastline was first discretised into shore-normal transects. Planview sandy shorelines, representative of the mean high-water line, were obtained from the Smartline dataset (Hazelwood, 2009). These shorelines were used to generate a series of *major* shore-normal transects spaced 100 m apart alongshore, aligning with the spatial resolution of a nearshore wave transformation tool used to predict nearshore wave conditions. Raw marine debris observations located within 50 m of each *major* transect were averaged (i.e., 100 m alongshore-average). This window aimed to minimise the localised effects from rip currents, bore merging and three-dimensional features such as beach cusps and rip horns (e.g., Harley et al., 2011b; Harley et al., 2015). Considering four storms, a total of 471 *TWL* observations corresponding to 345 *major* transects were used for comparison with predictions. These observations were subsequently used to analyse the intra-beach and regional variability in TWLs across the four storms. The final step in pre-processing marine debris observations for model-data comparison involved scaling the *TWL* observations associated with *R*_max_ to the equivalent elevation exceeded by 2% of the waves (i.e., *R*_2%_), which is the statistic that most runup formulae predict. Supplementary Appendix A shows a relationship between *R*_max_ and *R*_2%_ that is applied to the *TWL* observations prior to model-data comparison.

### *TWL* modelling

#### Modelling approach and runup formulae

TWLs are modelled using *TWL = SWL + R.* Observations of *SWL* were sourced from nearby tide gauges (Figure 3c, lower panels). At this wave-dominated region, the focus is on evaluating different empirical runup formulae and specifications of beach slope to improve *TWL* modelling. To achieve this, seven runup models were selected based on their relevance and usage in previous coastal inundation studies in SE Australia. These included two formulae that are commonly used in coastal hazards studies in SE Australia (Shand et al., 2011), including a laboratory-based model by Hedges and Mase (2004) and an older formula developed from a wide spectrum of beaches in NSW (Nielsen and Hanslow, 1991). In addition, the model-of-models by Atkinson et al. (2017), developed for SE Australian beaches during small to moderate wave conditions was considered, as well as two additional models (Holman, 1986; Vousdoukas et al., 2012) that performed equally well against the Atkinson et al. (2017) dataset. The final two models included the widely used generalised formula of Stockdon et al. (2006) and a recent machine learning model that comprised storm-data within their development (Power et al., 2019). The mathematical expressions of these formulae are presented in Table 1.

**Table 1.** Summary of empirical models for predicting R_2%_ used in this study Table 1. long description.

Authors	Empirical formula	Description
Holman (1986)	$R_{2\%}=0.83\tan\beta \sqrt{H_{o}L_{o}}+0.2H_{o}$	Site-specific. Field formula from the Duck research facility, NC, USA.
Nielsen and Hanslow (1991)	$R_{2\%}=1.98L_{R}$ $L_{R}=0.85\tan\beta \sqrt{H_{o}L_{o}};\tan\beta \geq 0.1$ $L_{R}=0.085\sqrt{H_{o}L_{o}};\tan\beta <0.1$	Regional specific. Field formula from several beaches in SE Australia.
Hedges and Mase (2004)	$R_{2\%}=1.49\tan\beta \sqrt{H_{o}L_{o}}+0.34H_{o}$	Laboratory formula
Stockdon et al. (2006)	$R_{2\%}=0.043\sqrt{H_{o}L_{o}}$ ; $\tan\beta /\sqrt{H_{o}/L_{o}}$ < 0.3 $R_{2\%}=1.1\left(\sqrt{\frac{H_{o}L_{o}\left(0.563\beta^{2}+0.004\right)}{2}}+0.35\beta \sqrt{H_{o}L_{o}}\right);$ $\tan\beta /\sqrt{\frac{H_{o}}{L_{o}}}\geq 0.3$	Generalised formula from 10 field studies.
Vousdoukas et al. (2012)	$R_{2\%}=0.53\beta \sqrt{H_{o}L_{o}}+0.58\tan\beta H_{o}+0.45$	Site specific. Field formula from a steep beach in Algarve, Portugal
Atkinson et al. (2017)	$R_{2\%}=0.92\tan\beta \sqrt{H_{o}L_{o}}+0.16H_{o}$	Regional specific. Model-of-models (M2 in Atkinson et al., 2017) applied to SE Australian field data
Power et al. (2019)	Refer to Eq. 9 in Power et al. (2019)	Generalised machine learning formula based on a Gene-Expression Programming. Forced by $H_{o},L_{o}$ and *d_50_* (median sand grain size)*_._* A value of *d_50_* = 0.3 mm was used throughout this work, representative of sediment characteristics of New South Wales beaches.

Runup formulae are forced with offshore wave data inputs (*H_o_* and *L_o_*). Available wave data from offshore wave buoys (Figure 3c, upper panels) were first transformed to the 10 m contour to account for wave transformation across the continental shelf and intra-beach gradients in wave energy due to protruding headlands (Fellowes et al., 2022). A model (Baird, 2017) based on the WAVEWATCHIII v4.18 model (Tolman, 2014) was used to simulate wave conditions at 10 m depth contour every 100 m along the coastline, commensurate with the locations of 100 m spaced *major* shore-normal transects (Section “Transect dataset and marine debris observations pre-processing”). This tool utilises pre-developed transformation matrices (look-up tables) covering the full range of wave periods, directions and wave heights (19×19×19 joint combinations) pertinent to SE Australia. Depending on the location of the desired wave modelling output, the tool is forced with either the Sydney or Port Kembla wave buoy data (Figure 3a). Previous evaluations of this tool showed a strong agreement (Pearson correlation coefficient, *ρ* ~ 0.9) with nearshore wave buoys at two beaches considered in the present study (Baird, 2017; Kinsela et al., 2024). Following the recommendations by Stockdon et al. (2006) and Gomes da Silva et al. (2020), offshore storm data were transformed to 10 m depths and subsequently reverse-shoaled to offshore conditions (*H_o_, L_o_*) to comply with the input requirements of the selected runup formulae.

#### Beach slopes (*β)* from LiDAR and satellites

Adequate beach slope specification is critical for modelling coastal inundation hazard (e.g., Figure 2). This study leverages emerging remote-sensing technologies to derive historical beach slope magnitudes and their variability. A total of three beach slope proxies from LiDAR and satellite data were used for this purpose.

##### LiDAR data

Topographic data in the form of digital elevation models from multiple episodic regional scale LiDAR surveys commissioned by the NSW Government were used to estimate historical beach slope magnitudes and variability. On average, each beach was surveyed eight times (standard deviation ~3) over a 17-year period (2007–2023). Previous comparisons of LiDAR-derived topography and in situ RTK-GPS surveys in this region indicated vertical and horizontal accuracies better than 0.3 and 0.8 m, respectively (Middleton et al., 2013; Doyle and Woodroffe, 2018). LiDAR typically covers the entire dune-beach system down to MSL (~0 m AHD). The reader is referred to Beuzen et al. (2019), Doyle and Woodroffe (2018), Harley (2017), Linklater et al. (2023) and Middleton et al. (2013) for more details.

Following the discretisation of the coastline into 100 m *major* transects (Section “Transect dataset and marine debris observations pre-processing”), historical (i.e., long-term average) beach slopes at these locations were calculated from *β* distributions sampled over different LiDAR flights and 100 m alongshore windows. Similar to Doran et al. (2015), an additional higher resolution 10 m spaced *minor* transect dataset was used to sample *β* over 100 m windows (i.e., 11 transects spaced every 10 m), capturing alongshore variability in *β* associated with beach cusps and rip horns (Harley et al., 2011b; Harley et al., 2015; Ibaceta et al., 2023).

Individual *β* samples were estimated using a linear regression between fixed upper and lower elevations. Based on previous literature that specified elevations from morphological and water level proxies (e.g., dune crest, berm height, tidal planes, Doran et al., 2015; Stockdon et al., 2023; Vos et al., 2020), this study explores two approaches: (1) beach slopes calculated between MSL (~0 m AHD) and the MHWS (mean high water springs, 0.7 m AHD this site, Beuzen et al., 2019) and (2) beach slopes calculated between MSL and the berm height (e.g., Harley et al., 2015). These two proxies represent (1) the slope of the partial inter-tidal zone (hereafter MSL-MHWS) and (2) the beach steepness considering the largely subaerial beach face morphology at these intermediate beaches that would be in the swash zone at high tides (hereafter MSL-berm). The 2 m AHD contour was selected as a pragmatic estimate of the beach berm height across this region as recommended by Kinsela et al. (2017).

##### Satellite-derived slopes

Given the limited availability of continuous high-resolution LiDAR data elsewhere, a novel satellite-derived *β* dataset was also tested for estimating extreme *TWL.* The publicly available Australian beach slope dataset presented in Vos et al. (2022) was employed for this purpose. In this dataset, beach slopes were estimated using the CoastSat Toolbox (Vos et al., 2019). CoastSat retrieves time series of cross-shore shoreline evolution from 30+ years (1987 onwards) of publicly available satellite imagery (Landsat 5, 7, 8 and Sentinel-2). By analysing these time series at individual shore-normal transects, the long-term partial inter-tidal beach slope (i.e., the slope between MSL and MHWS) is estimated using a frequency-domain analysis (Vos et al., 2020), such that an optimised *β* minimises high-frequency tidal variations in the satellite-derived signal. This method demonstrated strong accuracy (coefficient of determination, *ρ^2^* ~ 0.9) between satellite-derived slopes (hereafter CoastSat) and in situ long-term averages across eight different beaches in Australia, Europe, USA and Mexico (Vos et al., 2020). However, slightly more scatter was observed at intermediate beach states characterised by larger temporal variability in beach slopes, compared to better performance at gently sloping profiles. The dataset provides *β* magnitudes every 100 m along the coastline, consistent with the resolution of the *major* transect dataset (Section “Transect dataset and marine debris observations pre-processing”).

#### Performance of *TWL* modelling

Hourly time series of *TWL = SWL + R* were modelled using observed *SWL* data, local nearshore wave data, seven runup formulae and three *β* specifications (two from LiDAR and one from CoastSat). Maximum hourly values of *TWL* during the storm were evaluated against 471 post-processed *TWL* debris lines observations collected from 40 beaches during four storms. Model accuracy was assessed using several metrics, including the coefficient of determination (*ρ^2^*), root mean square error (*RMSE*) and model bias (*BIAS).*

## Results

### Observations of extreme *TWLs*

**Figure 4.**
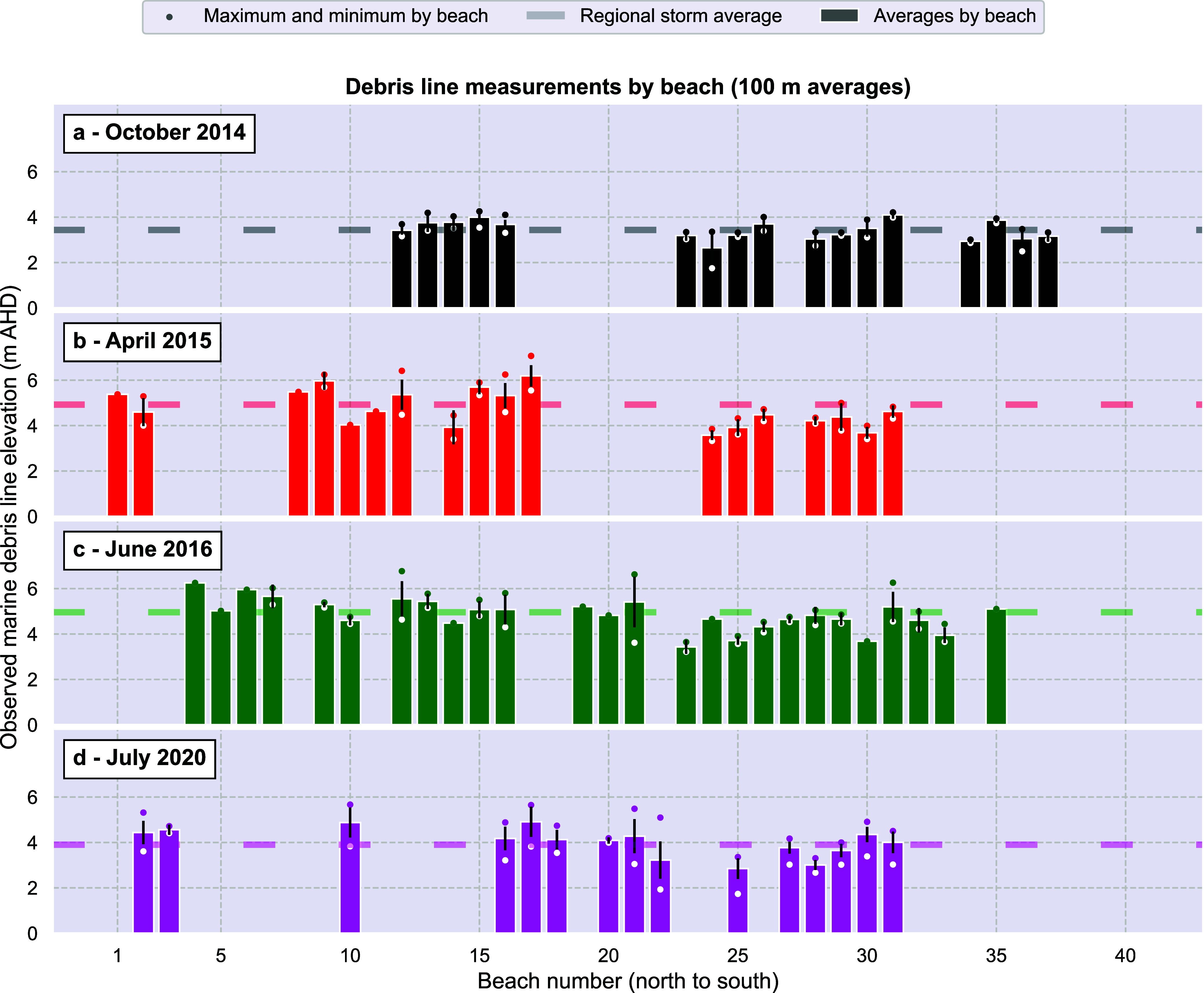
Regional observations of total water levels from marine debris lines recorded during four storms (panels a–d) across 37 individual beaches (ordered from north to south, horizontal axes). Horizontal dashed lines indicate the regional average marine debris elevation by storm. Bars show the intra-beach average and standard deviation (black lines). Coloured and white dots indicate the maximum and minimum measurements by beach, respectively. No bars indicate no measurement at that beach. Figure 4. long description.

Figure 4 illustrates the regional variability in observed extreme *TWL* during four storms in SE Australia, referencing data to the local Australian vertical datum (m AHD). These results summarise 100 m averaged marine debris observations, commensurate with the resolution of *major* transect dataset. From top to bottom, panels a–d show results across four storms (October 2014–July 2020). From left to right, *TWL* data (in bars) denote averages by beach, ordered from north to south (Beach #1 to #40, horizontal axes). Maxima and minima *TWL* along individual beaches are shown in coloured and white dots, respectively. The error bars reflect intra-beach variability, calculated as the standard deviation of *TWL* observations.

Across the four storms, the June 2016 event exhibited the highest average regional *TWL* at 5.2 m AHD, closely followed by the April 2015 storm with an average of 5.1 m. The July 2020 event recorded a lower average *TWL* of 4.7 m AHD, while the October 2014 event had the lowest average of 3.7 m AHD. *TWL* varied along individual beaches (error bars, standard deviation) by up to 1.1 m (minimum of 0.1 m). The distinction between maximum and minimum *TWL* at each beach (coloured and white dots) highlights localised effects and alongshore variability, with the maximum observations exceeding the minimum by an average of 0.7 m, ranging from 0.1 to 3.2 m across the dataset. These results suggest that the observed local and regional scale variability in extreme *TWLs* may arise from differing magnitudes of local wave characteristics and beach morphology, which are critical factors to consider in modelling approaches.

### Modelling extreme *TWLs*

**Figure 5.**
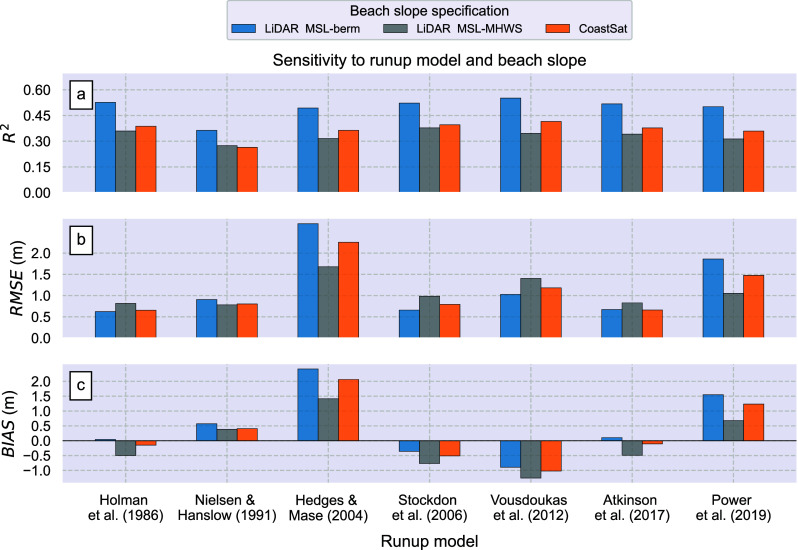
Summary statistics of total water level modelling (TWL = SWL + R, 472 observations) for three different beach slope specifications (indicated by different colours) and seven runup formulae (displayed on horizontal axes) across all four storms. Top to bottom panels show the coefficient of determination (ρ^2^), root mean square error (RMSE) and BIAS. Different runup formulae are ordered chronologically from left to right. Figure 5. long description.

Figure 5 summarises the performance of various empirical approaches in predicting *TWL*, using combinations of measured *SWL*, local nearshore wave data, seven runup models and three beach slope specifications. Model skill was calculated for the entire dataset (471 observations) after scaling *TWL* to the elevation exceeded by 2% of the waves (Supplementary Appendix A, hereafter referred to as *TWL* for simplicity). From top to bottom, panels (a)–(c) in Figure 5 depict the coefficient of determination *ρ^2^* (−)*, RMSE* (in m) and *BIAS* (in m), respectively. The horizontal axis indicates different runup models, ordered chronologically from left to right, with coloured bars denoting different beach slope specifications. Blue and grey bars correspond to LiDAR-derived beach slopes calculated between the lower MSL and the upper berm (2 m AHD, MSL-berm, average slopes 0.08 and std. 0.02) and MHWS (0.7 m AHD, MSL-MHWS, average slopes 0.06 and std. 0.02), respectively. Orange bars show results for the CoastSat beach slopes (MSL-MHWS, average slopes 0.07 and std. 0.01).

Figure 5a reveals that using beach slopes between the MSL and the berm (MSL-berm, blue bars) explained up to 57% of the *TWL* variability (*ρ^2^* = 0.57, Vousdoukas et al., 2012 model), and on average, 51% of variability across all models. Model skill decreases for *β* calculated in the partial intertidal zone (MSL-MHWS) and CoastSat (*ρ^2^* = 0.36 and 0.38 across all models, respectively). The differences in model performance using different beach slope specifications were less remarkable in terms of *RMSE* (Figure 5b) and *BIAS* (Figure 5c), although variability across different runup formulae was notable (horizontal axes). RMSE values were generally lower than 1 m, with the models by Holman (1986), Stockdon et al. (2006) and Atkinson et al. (2017) achieving the lowest errors (~0.6 m). Conversely, the formulae by Hedges and Mase (2004) and Power et al. (2019) exhibited lower performance (RMSE >1.1 m and up to 2.7 m). As for the *BIAS* metric (Figure 5c, *BIAS* = predicted – observed), Hedges and Mase (2004) and Power et al. (2019) models showed considerable overestimation, up to 2.5 and 1.6 m, respectively. Modelling *TWL* using other formulae resulted in both over-and-underestimations of less than ~1 m. Notably, the models by Atkinson et al. (2017) and Holman (1986) demonstrated the lowest *BIAS* (|*BIAS*| < 0.4 m) across all beach slope specifications.

**Figure 6.**
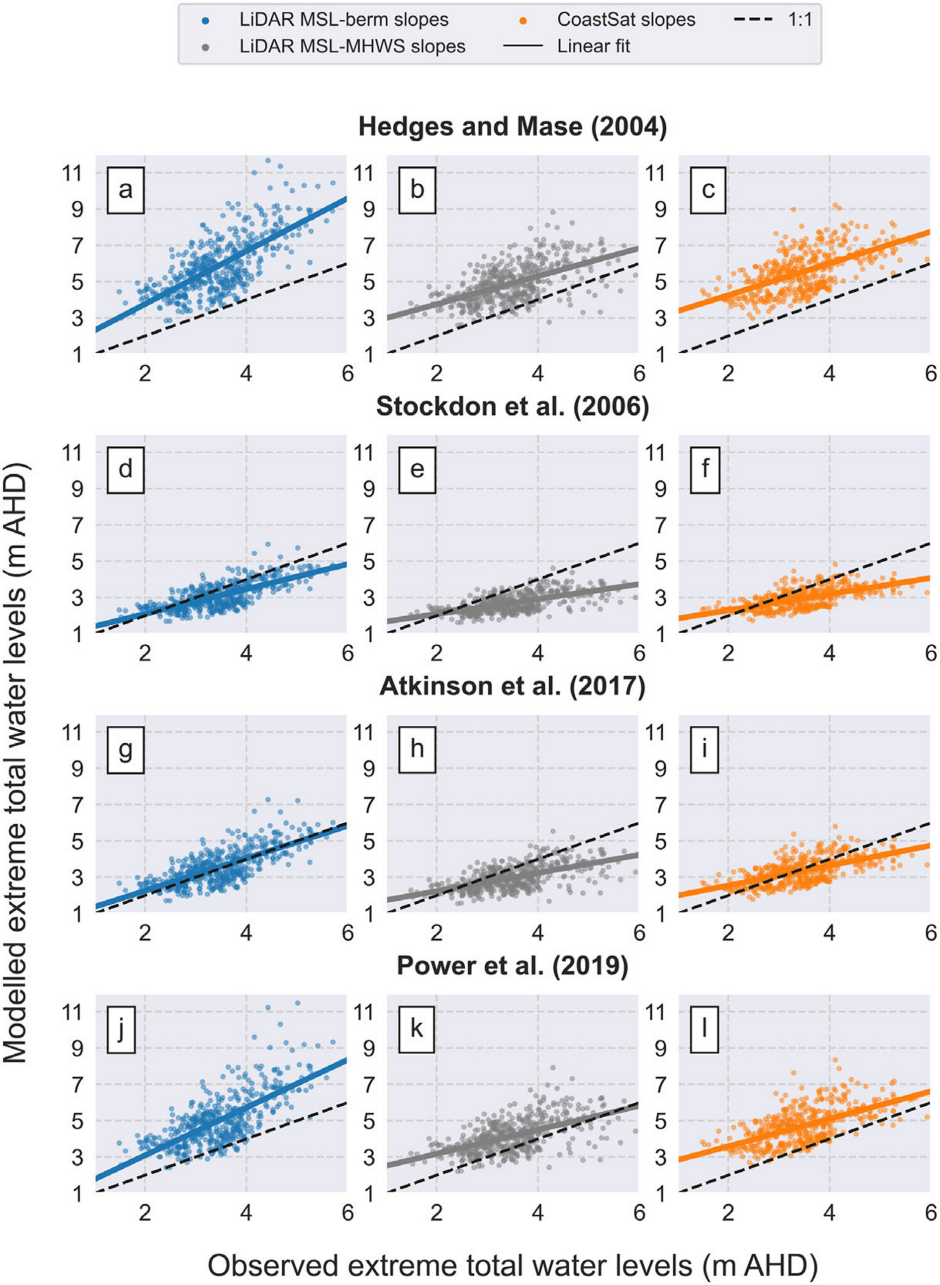
Example scatter plots of total water level modelling (TWL = SWL + R, 471 observations) for three different beach slope specifications (represented by different colours from left to right) and four runup formulae (displayed from top to bottom panels). Each dot indicates an individual observation and its corresponding modelled value. The black dashed lines present a perfect fit (1:1). Coloured solid lines are linear fits obtained from the modelled data and are included to facilitate the interpretation of the model’s skill and performance. Figure 6. long description.

To explore the results in further detail, representative scatter plots of modelled and observed *TWL* are presented in Figure 6 for a mix of laboratory, site specific, generalised and machine learning runup formulae. Left to right panels indicate different *β* specifications (from left to right, LiDAR MSL-berm, LiDAR MSL-MHWS and CoastSat). Top to bottom panels indicate different runup formulae, including a laboratory-based model (Hedges and Mase, 2004), the widely used and generalised model by Stockdon et al. (2006), the SE Australian model-of-models by Atkinson et al. (2017) and a recent machine learning model by Power et al. (2019). Additional scatter plots for the remaining three models used in the present study can be found in Supplementary Appendix B. Black dashed lines indicate a 1:1 line (perfect fit), while continuous coloured lines indicate the modelled linear fit to assist with the interpretation of results.

The scatter plots show varying degrees of model performance, with individual errors ranging from ~1 m and up to 2–3 m. The Hedges and Mase (2004) and Power et al. (2019) (Figure 6a–c, j–l) tend to overpredict *TWL* across all beach slope specifications, as indicated by their linear fits lying above the 1:1 line. In contrast, the models by Stockdon et al. (2006) (Figure 6d–f) and Atkinson et al. (2017) (Figure 6g–i) provide predictions closer to the 1:1 line. This is particularly true for the combination of MSL-berm slopes (blue dots) and the Atkinson et al. (2017) formula (Figure 6g, *ρ^2^ =* 0.55, *RMSE* = 0.6 m, *BIAS* = 0.1 m) that resulted in superior predictions over the entire range of *TWL* values including extremes (e.g., *TWL* > 4 m AHD). In contrast, predictions using the Stockdon et al. (2006) formula were less evenly distributed around the 1:1 line and underpredicted *TWL* extremes (Figure 6d). Underprediction of extreme *TWL* is also apparent for *β* specifications in the partial intertidal zone (MSL-MHWS) and CoastSat (Figure 6e,f,h,i), suggesting that considering as much of the entire beach face as practical (MSL-berm) is critical for predicting extreme *TWL* during storms.

## Discussion

### Modelling extreme *TWLs*

#### Application of runup formulae to *TWL* modelling

Coastal practitioners often face the challenge of selecting from numerous existing (30+) and emerging runup formulae for coastal inundation risk assessments. Comparing 471 *TWL* observations with models using gauged *SWL* and seven runup formulae resulted in varying degrees of model performance (Figure 5). The most accurate models were the SE Australia-specific Atkinson et al. (2017) and Holman (1986), achieving the lowest *RMSE* (~0.6 m and minimal average errors *(|BIAS| <* 0.4 m*)* across all beach slope specifications. Although the Holman (1986) formula was calibrated with data from the Duck Research Facility (USA), Atkinson et al. (2017) attributed their comparable performance to an identical mathematical structure and similar magnitudes in their model’s free-parameters (see Table 1). It is plausible that the strong performance of the Holman (1986) model reflects similarities between the intermediate beach states typical of SE Australia and those observed at Duck.

Notably, this study demonstrated that the most accurate model, Atkinson et al. (2017), which was developed during small to moderate conditions (*H_o_ ~* 1.5 m in their study), can be extrapolated to extreme conditions (*H_o_ ~* 6 to 8.1 m in this study) without loss of performance. This result is relevant since accurate *TWL* predictions during extreme storms are likely the most important to coastal practitioners in identifying low-lying areas subjected to coastal flooding (Almar et al., 2021). The feasibility of transferring other formulae to predict extreme conditions elsewhere requires further validation.

Modelling *TWL* using other runup models formulated elsewhere (e.g., Holman, 1986; Stockdon et al., 2006; Vousdoukas et al., 2012) resulted in RMSE errors of ~1 m (Figure 5b), except for the laboratory-based models (Hedges and Mase, 2004) and machine learning applications (Power et al., 2019), which exhibited higher errors (RMSE = 1.1–2.7 m) and overestimated *TWL* by up to 2.5 m (Figure 5c). These results are consistent with previous research indicating that laboratory-derived formulae tend to underperform when applied to field-data (Atkinson et al., 2017) and ‘generalised’ machine learning approaches can produce significant errors when used outside their learning/training range (Goldstein et al., 2019; Beuzen and Splinter, 2020; Gomes da Silva et al., 2020; Kim and Lee, 2024). The widely adopted formula by Stockdon et al. (2006) consistently underestimated *TWL* (*BIAS* approximately *−*0.5 m across all beach slope specifications), particularly at the most relevant higher levels (e.g., *TWL >* 4 m, Figure 6d–f). Consistent with previous studies (Vousdoukas et al., 2012; Stockdon et al., 2014; Atkinson et al., 2017), systematic errors in generalised ‘one-model-fits-all’ parameterisations, such as Stockdon et al. (2006), can lead to inaccuracies when applied to other sites. Novel approaches that tailor machine-learning model architectures to varying hydrodynamic and beach-state conditions (e.g., Itzkin et al., 2025) provide a more suitable solution than generalised, one-model-fits-all frameworks.

Importantly, this study demonstrated that for the wave-dominated SE Australian coastline, models previously calibrated for similar morphodynamic characteristics (Atkinson et al., 2017) performed better (including during extreme storms) than generalised, emergent machine learning and laboratory-based approaches. These results imply that coastal practitioners are recommended to increasingly utilise site-specific formulae in their coastal risk assessments. Depending on project/management objectives, first-pass assessments of coastal inundation risk employing appropriate *TWL* modelling approaches can be complemented by more advanced process-based modelling methodologies (e.g., Barnard et al., 2019b; Zornoza-Aguado et al., 2024). The success of these methodologies will only be achieved if data collection efforts during storms (Kim et al., 2023) continue to support runup benchmarking studies (e.g., Atkinson et al., 2017; Gomes da Silva et al., 2020).

#### Sensitivity of beach slope specifications to *TWL* modelling

Defining appropriate beach slope specifications (*β)* is crucial for coastal inundation risk assessments (e.g., Figure 2, Aucan et al., 2019; Kim et al., 2023; Kim and Lee, 2024). To assist coastal practitioners in SE Australia with a fit-for-purpose quantification of this key parameter, our study tested three time-invariant historical beach slope specifications from emerging remote sensing technologies. *TWL* predictions using long-term LiDAR slopes calculated between the MSL and berm height (*ρ* ^2^ = 0.51 across all models) outperformed those using slopes calculated in the partial intertidal zone (LiDAR, MSL-MHWS) and satellite-derived CoastSat slopes, which also represent the MSL-MHWS, by approximately 15% (*ρ^2^* = 0.36 and 0.38, respectively, Figure 5a).

LiDAR-derived slopes are better (or at least similarly well) suited for coastal inundation risk assessments than satellite-derived approaches for storm conditions in this coastline. Comparable skill between LiDAR MSL-MHWS and CoastSat slopes is not surprising, as the latter are also a proxy of the partial intertidal zone (MSL-MHWS) (Vos et al., 2022, 2020). Both MSL-MHWS specifications showed underestimation of *TWL* extremes (Figure 6h,i), highlighting the importance of using as much of the beach face as practical (MSL-berm) for predicting extreme *TWL* during storms in intermediate-state beaches common in SE Australia (Wright and Short, 1984). These results highlight the importance of using a broader portion of the beach face (here, the MSL–berm zone) to estimate *TWL* under storm conditions, rather than relying on narrower segments (e.g., only part of the intertidal zone) that may be more suitable for calmer conditions.

To our knowledge, this study is the first published attempt to implement and evaluate satellite-derived slopes into large-scale *TWL* modelling. Although these slopes resulted in lower (or similar) skill than higher resolution LiDAR slopes, they remain a valuable resource for data-poor regions lacking continuous coastal monitoring programs using more costly techniques (Splinter et al., 2018; Vitousek et al., 2023). The emergence of enhanced higher-resolution satellite-derived techniques (e.g., CubeSats, Doherty et al., 2022) will potentially result in improved quantifications of satellite-derived coastal morphological variables from local to global scales (e.g., Pronk et al., 2024) and pave the way for enhanced local to global coastal inundation risk assessments in view of accelerating SLR.

### Challenges and opportunities for coastal inundation risk studies

#### Leveraging beach slope uncertainty for *TWL* modelling

Despite decades of research on runup modelling, there remains no perfect predictive runup model for coastal inundation risk studies. As demonstrated in this study, even the best performing formulae and beach slope specifications can lead to scatter and large individual errors of several metres (e.g., Figure 5b). Instead of solely aiming to reduce these errors in deterministic models (e.g., Kim and Lee, 2024), emerging and growing research on probabilistic predictions offers a more comprehensive approach to coastal hazard modelling by predicting a range of possible outcomes (Kinsela et al., 2017; Limber et al., 2018; Beuzen et al., 2019; Vitousek et al., 2021). Essentially, probabilistic outputs provide likelihoods of *TWL* which are generally required for risk assessments by coastal management and planning frameworks.

**Figure 7.**
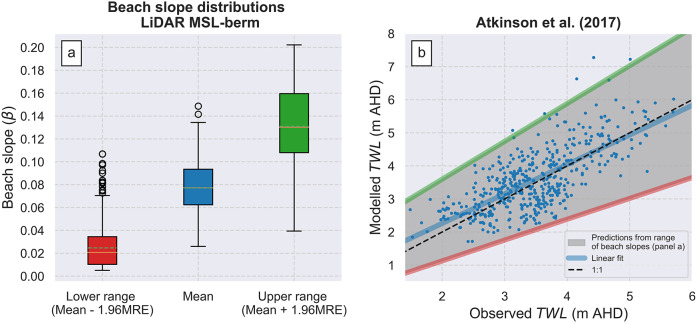
Influence of varying beach slope ranges on predicting the highly variable observed total water levels. (a) Lower, mean and upper range distributions of beach slopes derived from spatio-temporal LiDAR data. While the above analyses considered the regional distribution of averaged beach slopes across each transect (central boxplot in panel a), an updated analysis considering beach slope uncertainty accounts for the majority of observed range in total water levels (panel b). Figure 7. long description.

To enhance *TWL* forecasting performance, we explored a straightforward probabilistic, data-driven approach that leverages the uncertainty inherent in available beach slope data, disregarding other sources of uncertainty that cannot be quantified for this study (e.g., nearshore wave transformation). Considering that average beach slopes were calculated from several LiDAR flights centred at 100 m spaced *major* transects (e.g., Doran et al., 2015, Section “Transect dataset and marine debris observations pre-processing”), the *β* range can be used to estimate a wider range of *TWL* outcomes (e.g., Figure 2). Doran et al. (2015) defined the concept of mean residual error, MRE (i.e., the standard deviation of *β* at each transect) and used it to estimate lower and upper beach slope ranges (95% C.I., Mean ± 1.96MRE, here shown in Figure 7a for MSL-berm slopes). By repeating the *TWL* modelling process with these upper (green) and lower (red) *β* bounds, a range of *TWL* predictions were obtained that account for beach slope variability (Figure 7b). This straightforward analysis, previously applied in Stockdon et al. (2023), considers the variability in beach slope, as the only single source of uncertainty and shows that the range of *TWL* predictions (between green and red linear regressions) encompass the majority of the model errors in deterministic models from average beach slopes (blue dots) for this coastline. This assessment demonstrates the potential to leverage the uncertainty of this key input variable (*β)* as a source of uncertainty for *TWL* modelling, giving practitioners a simple means to quantify uncertainty without incorporating less-understood errors from sources such as formulas, wave transformation or wave-parameter estimates.

By implementing probabilistic *TWL* modelling approaches, coastal practitioners will be able to provide more comprehensive assessments of coastal flooding considering a complete range of potential modelling outcomes now and into the future.

#### Implications for future coastal inundation hazard and risk assessments

*TWL* forecasts are crucial to identify areas of coastline that may experience coastal flooding in coming decades. Under the well-established assumption of minor (5%–10%) projected changes in global waves (Morim et al., 2019; Casas-Prat et al., 2024), future open-coast inundation assessments typically incorporate magnitudes of SLR directly into *TWL* models (Vitousek et al., 2017; Almar et al., 2021). SLR projections vary based on different time horizons, climate change and emission scenarios, currently known as Shared Socioeconomic Pathways (SSPs).

**Figure 8.**
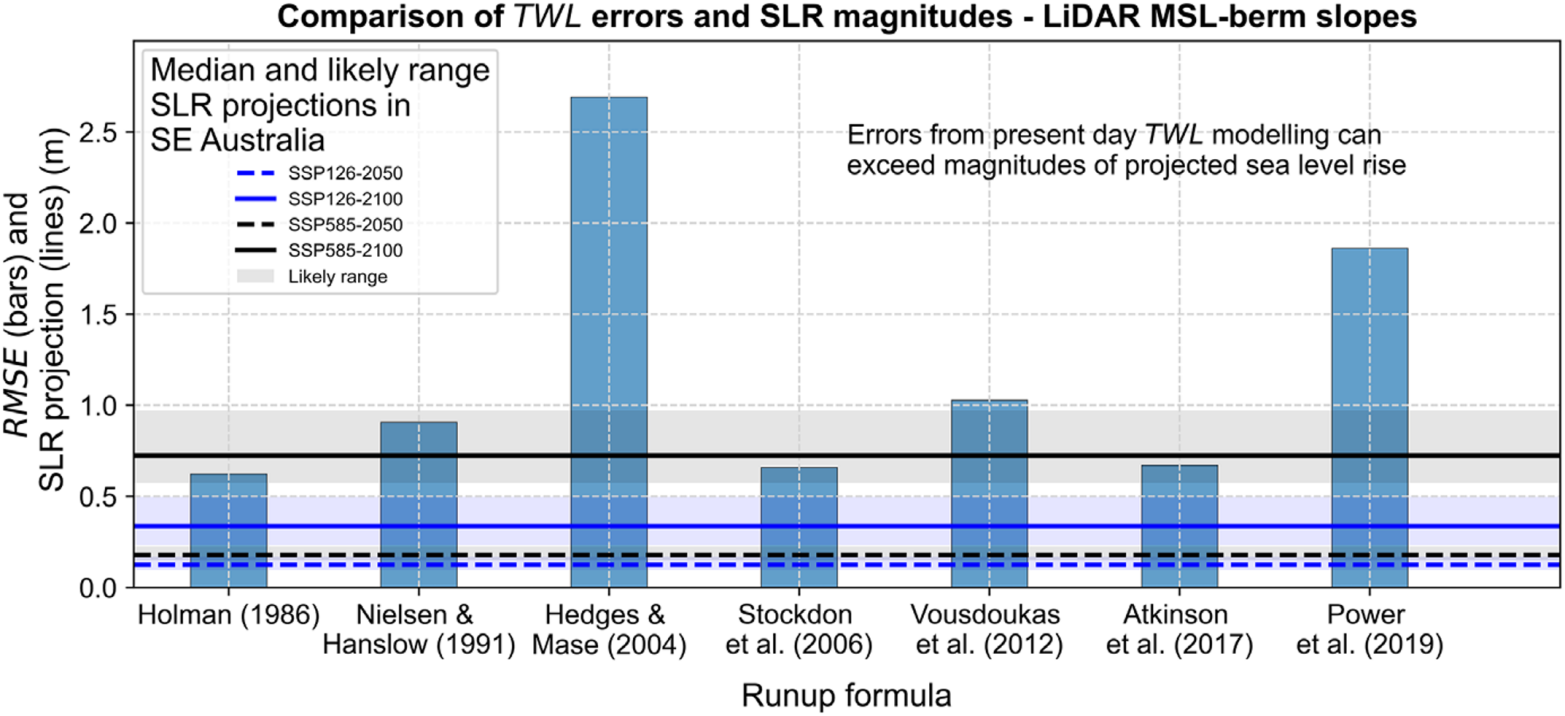
Implications for future coastal inundation risk studies. Comparison of the TWL modelling skill (RMSE) for different formulae and projected sea level rise magnitudes in SE Australia by 2050 and 2100 under SSP126 and SSP585 scenarios. Figure 8. long description.

Given the observed errors in current *TWL* predictions (e.g., Figure 5a), it is worthwhile to compare these with the uncertainties in future SLR projections (e.g., Wahl et al., 2017; Agulles and Jordà, 2024). Figure 8 shows the *TWL* modelling errors (RMSE) from different runup formulae with the median and likely range (17%–83%) projected SLR for 2050 and 2100 in Sydney (SE Australia), using the latest data from the Intergovernmental Panel on Climate Change Sixth Assessment Report (IPCC, 2023; Kopp et al., 2023). This analysis reveals that the uncertainties in present-day *TWL* modelling can exceed both the magnitudes and uncertainties of projected SLR across the broad spectrum of plausible scenarios (i.e., SSP126 to SSP585 scenarios, horizontal lines and shaded areas in Figure 8). This result highlights the need for improved *TWL* modelling and the incorporation of uncertainty across all constituents of the modelling framework (e.g., Figure 7). As the distributions of future sea level uncertainty up to 2150 and across different pathways become more accessible through publicly available resources (e.g., the NASA sea level projection tool, e.g., Garner et al., 2022, https://sealevel.nasa.gov/ipcc-ar6-sea-level-projection-tool), there is a significant opportunity to enhance the assessments of future open-coast inundation risks. With global coastal policies increasingly mandating the incorporation of climate change in future coastal planning (Thom, 2022), coastal practitioners will need to incorporate uncertainty across the entire modelling framework, including rising sea levels, potential changes in waves (Morim et al., 2023) and storm surges (Muis et al., 2023). Until we gain more holistic knowledge on these future drivers of coastal change, related planning decisions should be made with caution.

## Conclusions

Coastal practitioners increasingly require accurate predictions of extreme *TWL* in order to implement appropriate disaster risk measures and effectively prepare for coastal flooding events. This study has presented a novel, comprehensive and large-scale dataset of extreme *TWL* observations collected during four storms in SE Australia. Following the description of this dataset, this study performed a comprehensive analysis on the performance of different runup formulae and beach slope specifications in predicting observed *TWL.*

Analysis of 471 *TWL* observations from these storms revealed considerable intra-beach and regional variability in *TWL.* By comparing these observations with *TWL* models derived from gauged *SWL*, local nearshore wave transformation tools, seven runup formulae and three beach slope specifications from remote-sensing techniques, varying runup modelling performances were observed. *TWL* predictions using historical beach slopes from LiDAR data outperformed (~15% increase in model skill) the models using emerging satellite-derived slopes, although the latter are still deemed a reasonable/valuable source for data-poor regions. Results indicated varying model performances across different runup formulae and identified the best performance for a model previously developed in SE Australia under moderate wave conditions (Atkinson et al., 2017, ρ^2^ = 0.55, RMSE = 0.6 m, BIAS = 0.4 m). This highlights the effectiveness of using site-specific tailored models over more generic, widely adopted ‘one-model-fits-all’ approaches and emergent machine learning formulae. Furthermore, this study demonstrated that existing models typically calibrated during small to moderate wave conditions can be reliably extrapolated to extreme storm events, provided they are applied to sites of similar morphodynamic characteristics.

Our analyses showed that uncertainty in beach slope measurements could account for a large portion of the observed model errors, highlighting the potential to leverage the inherent uncertainty of this variable as a single major source of uncertainty in present-time *TWL* modelling. Overall, this work presented the value of emerging *TWL* datasets collected during storms and provided a robust framework to assist coastal practitioners in enhancing forward forecasting applications. By leveraging both site-specific models and uncertainty in key model input variables, coastal risk assessments can be improved, leading to better preparedness and response strategies in the face of increasingly extreme coastal events.

## Supporting information

10.1017/cft.2026.10043.sm001 Ibaceta et al. supplementary material Ibaceta et al. supplementary material

## Data Availability

Transect data are available at https://datasets.seed.nsw.gov.au/dataset/nsw-open-coast-inundation-from-wave-overtopping-2025.

## Supplementary material

The supplementary material for this article can be found at http://doi.org/10.1017/cft.2026.10043.

## Long descriptions

### Figure 1. Long description

The diagram shows a yellow sandy beach profile on the left and blue ocean water on the right. A horizontal dashed line at the bottom is labeled Datum. Moving vertically upward from the Datum, the water level is divided into two primary stacked segments.

1. The lower segment is Still water levels S W L, which is composed of three layers from bottom to top: Mean sea level, Non-tidal residuals e.g., ENSO, and Tide.

2. The upper segment is Wave runup R, which consists of two layers: Wave set-up and Swash at the top.

The combined height of S W L and R is labeled as T W L on the far left. On the right, where the water meets the shore, a black angle is labeled with the Greek letter beta, representing the beach slope. A large blue wave is shown breaking toward the shore, with the water extending up the slope of the dune to the highest point of the Swash layer.

Navigate back to Figure 1..

### Figure 2. Long description

Panel a, titled Narrabeen S E Australia Historical surveys, features a line graph with Chainage in meters on the x-axis from 0 to 150 and Elevation in meters on the y-axis from minus 2 to 10. A dashed horizontal line marks M S L at 0 meters. A thick black line represents a steep profile, while a dense cluster of thin grey lines shows the dynamic beachface between chainage 25 and 100, varying between elevation 4 and minus 1. An inset histogram shows counts on the y-axis and Beach slope beta on a logarithmic x-axis from 0.01 to 0.2. The distribution peaks around 0.1, with vertical dashed lines marking the 1 percent and 99 percent bounds.

Panel b, titled Influence of beach slope in runup estimations, plots Offshore wave height in meters from 3 to 8 on the x-axis against Runup in meters from 1.0 to 4.5 on the y-axis. A central thick black line labeled median shows a linear increase in runup from approximately 2.1 to 3.4 meters as wave height increases. This line is centered within a wide grey shaded area labeled 1-99 percent predictions from range of beach slopes. The shaded area expands as wave height increases, ranging from approximately 1.2 to 2.8 meters at an offshore wave height of 3, and from 1.9 to 4.5 meters at an offshore wave height of 8. A text box notes large variability in runup estimations for dynamic beachfaces.

Navigate back to Figure 2..

### Figure 3. Long description

Panel a is a geographical map of the Southeast Australian coast between Newcastle and Shoalhaven. Colored dots along the coastline indicate debris surveys: purple for July 2020, green for June 2016, red for April 2015, and black for October 2014. Yellow stars mark wave buoys and white triangles mark tide gauges near Sydney and Port Kembla. A dashed yellow line indicates the 70 meter depth contour.

Panel b is a photograph showing a dense line of dark marine debris on a sandy beach. An inset photo shows a researcher using an R T K G N S S pole for monitoring.

Panel c is a grid of 16 line graphs organized by four storm events (columns) and four metrics (rows). The x-axis for all graphs is Days from storm H sub s peak, ranging from minus 1 to 3.

* Row 1: Significant wave height H sub s in meters. Peaks reach approximately 6 meters in October 2014, 8 meters in April 2015, and 6 meters in June 2016 and July 2020.

* Row 2: Peak wave period T sub p in seconds, showing fluctuations between 6 and 14 seconds.

* Row 3: Peak direction D sub p in degrees, showing shifts in wave orientation during the storms.

* Row 4: Tide gauge water level in meters A H D, showing oscillating tidal patterns between minus 0.8 and 1.2 meters.

Solid lines represent Sydney data and dotted lines represent Port Kembla data. Vertical shaded bars indicate the timing of debris line surveys.

Navigate back to Figure 3..

### Table 1. Long description

The table contains three columns: Authors, Empirical formula, and Description.

* Holman 1986: R sub 2 percent = 0.83 tan beta square root of H sub o L sub o + 0.2 H sub o. Description: Site-specific field formula from Duck research facility, NC, USA.

* Nielsen and Hanslow 1991: R sub 2 percent = 1.98 L sub R. Where L sub R = 0.85 tan beta square root of H sub o L sub o if tan beta is greater than or equal to 0.1; and L sub R = 0.085 square root of H sub o L sub o if tan beta is less than 0.1. Description: Regional specific field formula from SE Australia.

* Hedges and Mase 2004: R sub 2 percent = 1.49 tan beta square root of H sub o L sub o + 0.34 H sub o. Description: Laboratory formula.

* Stockdon et al. 2006: R sub 2 percent = 0.043 square root of H sub o L sub o if tan beta / square root of H sub o / L sub o is less than 0.3. Otherwise, R sub 2 percent = 1.1 open parenthesis square root of numerator H sub o L sub o open parenthesis 0.563 beta squared + 0.004 close parenthesis all over 2 + 0.35 beta square root of H sub o L sub o close parenthesis if tan beta / square root of H sub o / L sub o is greater than or equal to 0.3. Description: Generalised formula from 10 field studies.

* Vousdoukas et al. 2012: R sub 2 percent = 0.53 beta square root of H sub o L sub o + 0.58 tan beta H sub o + 0.45. Description: Site specific field formula from Algarve, Portugal.

* Atkinson et al. 2017: R sub 2 percent = 0.92 tan beta square root of H sub o L sub o + 0.16 H sub o. Description: Regional specific model applied to SE Australian field data.

* Power et al. 2019: Refers to Equation 9 in the original source. Description: Generalised machine learning formula forced by H sub o, L sub o, and d sub 50 median sand grain size.

Navigate back to Table 1..

### Figure 4. Long description

A multi-panel bar chart with four stacked horizontal panels labeled a through d. The y-axis for all panels is Observed marine debris line elevation in meters A H D, ranging from 0 to 6. The x-axis represents Beach number from 1 to 40, ordered North to South.

* Panel a (October 2014, black bars): Data is concentrated between beaches 15 and 37. Elevations mostly hover around the regional storm average of approximately 3.5 meters.

* Panel b (April 2015, red bars): Data points are scattered across the North (beaches 2 to 4), Central (beaches 9 to 18), and South (beaches 24 to 31). The regional average is higher, near 5 meters.

* Panel c (June 2016, green bars): This panel has the highest data density, with measurements for almost all beaches from 4 to 35. Most bars are near the 5-meter regional average, with a peak near beach 4 exceeding 6 meters.

* Panel d (July 2020, magenta bars): Data is sparse, appearing at beaches 2, 3, 10, 16 to 18, 20 to 22, and 25 to 31. The regional average is approximately 4 meters.

Each bar includes a black vertical error bar representing standard deviation. A white dot at the bottom of the error bar indicates the minimum measurement, and a colored dot at the top indicates the maximum measurement. Horizontal dashed lines in the background of each panel represent the regional average for that specific storm event.

Navigate back to Figure 4..

### Figure 5. Long description

A multi-panel bar chart titled Sensitivity to runup model and beach slope. A legend at the top identifies three beach slope specifications: LiDAR M S L-berm (blue), LiDAR M S L-M H W S (grey), and CoastSat (orange).

All panels share a horizontal axis labeled Runup model with seven categories ordered chronologically: Holman et al. 1986, Nielsen and Hanslow 1991, Hedges and Mase 2004, Stockdon et al. 2006, Vousdoukas et al. 2012, Atkinson et al. 2017, and Power et al. 2019.

Panel a: Vertical axis is R super 2 ranging from 0.00 to 0.60. Blue bars consistently show the highest correlation, peaking near 0.55 for Vousdoukas et al. 2012. Grey and orange bars are lower, generally between 0.25 and 0.40.

Panel b: Vertical axis is R M S E in meters ranging from 0.0 to 2.0. Hedges and Mase 2004 and Power et al. 2019 show the highest error, with blue bars exceeding 1.5 meters. Other models maintain R M S E below 1.0 meter.

Panel c: Vertical axis is B I A S in meters ranging from -1.0 to 2.0. A horizontal zero line indicates neutral bias. Hedges and Mase 2004 and Power et al. 2019 show strong positive bias (overestimation), while Stockdon et al. 2006 and Vousdoukas et al. 2012 show negative bias (underestimation) across all slope types.

Navigate back to Figure 5..

### Figure 6. Long description

A grid of twelve scatter plots arranged in four rows and three columns.

Axes and Legend:

* The horizontal x-axis represents Observed extreme total water levels in meters A H D, ranging from 1 to 6.

* The vertical y-axis represents Modelled extreme total water levels in meters A H D, ranging from 1 to 11.

* A top legend identifies three data series: blue dots for L i D A R M S L-berm slopes, grey dots for L i D A R M S L-M H W S slopes, and orange dots for CoastSat slopes.

* A black dashed line in every plot represents a 1 to 1 perfect fit.

* Solid colored lines represent the linear fit for each dataset.

Row-by-Row Breakdown:

* Row 1 (Hedges and Mase 2004): Panels a, b, and c. Data points are clustered above the 1 to 1 line, showing a significant overestimation of water levels. The linear fit lines have steep positive slopes.

* Row 2 (Stockdon et al. 2006): Panels d, e, and f. Data points are clustered below the 1 to 1 line, indicating an underestimation. The linear fit lines have a shallower slope compared to the first row.

* Row 3 (Atkinson et al. 2017): Panels g, h, and i. Data points are more closely aligned with the 1 to 1 dashed line, showing improved model accuracy, though some underestimation persists at higher values.

* Row 4 (Power et al. 2019): Panels j, k, and l. Similar to the first row, these plots show an overestimation, with data points and solid linear fit lines positioned above the dashed 1 to 1 line.

Navigate back to Figure 6..

### Figure 7. Long description

Panel a is a boxplot titled Beach slope distributions L i D A R M S L-berm. The y-axis is Beach slope beta ranging from 0.00 to 0.20. The x-axis has three categories. First, Lower range Mean minus 1.96 M R E shown as a red box with a median near 0.02. Second, Mean shown as a blue box with a median near 0.08. Third, Upper range Mean plus 1.96 M R E shown as a green box with a median near 0.13.

Panel b is a scatter plot titled Atkinson et al. 2017. The x-axis is Observed T W L in meters A H D ranging from 2 to 6. The y-axis is Modelled T W L in meters A H D ranging from 1 to 8. Blue data points are scattered around a central dashed black 1 to 1 line. A thick blue line represents the linear fit. A large grey shaded region, bounded by a green line at the top and a red line at the bottom, represents predictions from the range of beach slopes shown in panel a. The grey region encompasses the vast majority of the observed data points.

Navigate back to Figure 7..

### Figure 8. Long description

The X-axis is labeled Runup formula and lists seven specific models: Holman 1986, Nielsen & Hanslow 1991, Hedges & Mase 2004, Stockdon et al. 2006, Vousdoukas et al. 2012, Atkinson et al. 2017, and Power et al. 2019. The Y-axis is labeled R M S E (bars) and S L R projection (lines) in meters, ranging from 0.0 to 2.5.

Four horizontal lines represent S L R projections:

* S S P 1 2 6-2050: Dashed blue line at approximately 0.15 meters.

* S S P 5 8 5-2050: Dashed black line at approximately 0.2 meters.

* S S P 1 2 6-2100: Solid blue line at approximately 0.35 meters.

* S S P 5 8 5-2100: Solid black line at approximately 0.75 meters.

Shaded regions indicate the likely range for these projections, with a light blue band for 2050 scenarios and a light gray band for 2100 scenarios.

Vertical blue bars represent the R M S E for each formula:

* Holman: ~0.6 meters.

* Nielsen & Hanslow: ~0.9 meters.

* Hedges & Mase: ~2.7 meters (the highest error).

* Stockdon et al.: ~0.65 meters.

* Vousdoukas et al.: ~1.0 meter.

* Atkinson et al.: ~0.65 meters.

* Power et al.: ~1.85 meters.

Text in the upper right quadrant states: Errors from present day T W L modelling can exceed magnitudes of projected sea level rise.

Navigate back to Figure 8..

## References

[r1] Agulles M and Jordà G (2024) Quantification of error sources in wave runup estimates on two Mediterranean sandy beaches. Coastal Engineering 187, 104402. 10.1016/j.coastaleng.2023.104402.

[r2] Almar R, Ranasinghe R, Bergsma EWJ, Diaz H, Melet A, Papa F, Vousdoukas M, Athanasiou P, Dada O, Almeida LP and Kestenare E (2021) A global analysis of extreme coastal water levels with implications for potential coastal overtopping. Nature Communications 12(1), 3775. 10.1038/s41467-021-24008-9. PMC8213734 34145274

[r3] Almeida LP, Masselink G, Russell PE and Davidson MA (2015) Observations of gravel beach dynamics during high energy wave conditions using a laser scanner. Geomorphology 228, 15–27. 10.1016/j.geomorph.2014.08.019.

[r4] Atkinson AL, Power HE, Moura T, Hammond T, Callaghan DP and Baldock TE (2017) Assessment of runup predictions by empirical models on non-truncated beaches on the south-east Australian coast. Coastal Engineering 119, 15–31. 10.1016/j.coastaleng.2016.10.001.

[r5] Aucan J, Hoeke RK, Storlazzi CD, Stopa J, Wandres M and Lowe R (2019) Waves do not contribute to global sea-level rise. Nature Climate Change 9(1), 2. 10.1038/s41558-018-0377-5.

[r6] Baird (2017) NSW Coastal Wave Model State Wide Nearshore Wave Transformation Tool: Technical Report. Available at https://www.environment.nsw.gov.au/-/media/OEH/Corporate-Site/Documents/Research/Our-science-and-research/nsw-nearshore-wave-transformation-tool-report.pdf. Accessed 14 July 2026

[r7] Barnard PL, Erikson LH, Foxgrover AC, Hart JAF, Limber P, O’Neill AC, van Ormondt M, Vitousek S, Wood N, Hayden MK and Jones JM (2019a) Dynamic flood modeling essential to assess the coastal impacts of climate change. Scientific Reports 9(1), 4309. 10.1038/s41598-019-40742-z. 30867474 PMC6416275

[r8] Barnard PL, Erikson LH, Foxgrover AC, Hart JAF, Limber P, O’Neill AC, van Ormondt M, Vitousek S, Wood N, Hayden MK and Jones JM (2019b) Dynamic flood modeling essential to assess the coastal impacts of climate change. Scientific Reports 9(1), 4309. 10.1038/s41598-019-40742-z. 30867474 PMC6416275

[r9] Benito I, Aerts JCJH, Eilander D, Ward PJ and Muis S (2024) Stochastic coastal flood risk modelling for the east coast of Africa. npj Natural Hazards 1(1), 10. 10.1038/s44304-024-00010-1. 41675919 PMC12885944

[r10] Beuzen T, Goldstein EB and Splinter KD (2019) Ensemble models from machine learning: an example of wave runup and coastal dune erosion. Natural Hazards and Earth System Sciences 19, 2295–2309.

[r11] Beuzen T and Splinter K (2020) Machine learning and coastal processes. In Jackson DWT and Short AD (eds), Sandy Beach Morphodynamics. Oxford: Elsevier, pp. 689–710.

[r12] Blenkinsopp CE, Mole MA, Turner IL and Peirson WL (2010) Measurements of the time-varying free-surface profile across the swash zone obtained using an industrial LIDAR. Coastal Engineering 57(11-12), 1059–1065. 10.1016/j.coastaleng.2010.07.001.

[r13] Cariolet JM and Suanez S (2013) Runup estimations on a macrotidal sandy beach. Coastal Engineering 74, 11–18. 10.1016/j.coastaleng.2012.11.008.

[r14] Carrere L, Lyard F, Cancet M and Guillot A (2015) FES 2014, a new tidal model on the global ocean with enhanced accuracy in shallow seas and in the Arctic region. In EGU General Assembly Conference Abstracts, Vienna, Austria, 12–17 April, e5481. Available at https://ui.adsabs.harvard.edu/abs/2015EGUGA..17.5481C.

[r15] Cartwright DE and Longuet-Higgins MS (1956) The statistical distribution of the maxima of a random function. Proceedings of the Royal Society of London. Series A: Mathematical and Physical Sciences 237(1209), 212–232. 10.1098/rspa.1956.0173.

[r16] Casas-Prat M, Hemer MA, Dodet G, Morim J, Wang XL, Mori N, Young I, Erikson L, Kamranzad B, Kumar P, Menéndez M and Feng Y (2024) Wind-wave climate changes and their impacts. Nature Reviews Earth and Environment 5(1), 23–42. 10.1038/s43017-023-00502-0.

[r17] Chtioui T, Hakkou M, Aangri A, El Hassani F, El Mostafa Z and Benmohammadi A (2024) Assessing coastal flood risk under extreme events and sea level rise in the Casablanca-Mohammedia coastline (Morocco). Natural Hazards 120. 10.1007/s11069-024-06624-y.

[r18] Conlin MP, Cohn N and Adams PN (2023) Total water level controls on the trajectory of dune toe retreat. Geomorphology 438, 108826. 10.1016/j.geomorph.2023.108826.

[r19] Doherty Y, Harley MD, Vos K and Splinter KD (2022) A Python toolkit to monitor high-resolution shoreline change using Planetscope Cubesats. SSRN Electronic Journal 157, 105512. 10.2139/ssrn.4052360.

[r20] Doran KS, Long JW and Overbeck JR (2015) A Method for Determining Average Beach Slope and Beach Slope Variability for U.S. Sandy Coastlines.U.S. Geological Survey Open-File Report 2015-1053, Report: iv, 5 p.; Data Releases, 10.3133/ofr20151053. Accessed 14 July 2026.

[r21] Dowdy AJ, Pepler A, Di Luca A, Cavicchia L, Mills G, Evans JP, Louis S, McInnes KL and Walsh K (2019) Review of Australian east coast low pressure systems and associated extremes. Climate Dynamics 53(7-8), 4887–4910. 10.1007/s00382-019-04836-8.

[r22] Doyle TB, Hesp PA and Woodroffe CD (2024) Foredune morphology: Regional patterns and surfzone–beach–dune interactions along the New South Wales coast, Australia. Earth Surface Processes and Landforms 49(10), 3115–3138. 10.1002/esp.5879.

[r23] Doyle TB and Woodroffe CD (2018) The application of LiDAR to investigate foredune morphology and vegetation. Geomorphology 303, 106–121. 10.1016/j.geomorph.2017.11.005.

[r24] Fellowes TE, Vila-concejo A and Gallop SL (2019) Morphometric classification of swell-dominated embayed beaches. Marine Geology 411, 78–87. 10.1016/j.margeo.2019.02.004.

[r25] Fellowes TE, Vila-concejo A, Gallop SL, Harley MD and Short AD (2022) wave shadow zones as a primary control of storm erosion and recovery on embayed beaches. Geomorphology 399, 108072. 10.1016/j.geomorph.2021.108072.

[r26] Fritz HM, Petroff CM, Catalán PA, Cienfuegos R, Winckler P, Kalligeris N, Weiss R, Barrientos SE, Meneses G, Valderas-Bermejo C, Ebeling C, Papadopoulos A, Contreras M, Almar R, Dominguez JC and Synolakis CE (2011) Field Survey of the 27 February 2010 Chile tsunami. Pure and Applied Geophysics 168(11), 1989–2010. 10.1007/s00024-011-0283-5.

[r27] Garner GG, Hermans T, Kopp RE, Slangen ABA, Edwards TL, Levermann A, Nowicki S, Palmer MD, Smith C, Fox-Kemper B, Hewitt HT, Xiao C, Aðalgeirsdóttir G, Drijfhout SS, Golledge NR, Hemer M, Krinner G, Mix A, Notz D, Nurhati IS, Ruiz L, Sallée J-B, Yu Y, Hua L, Palmer T and Pearson B (2022) IPCC AR6 Sea Level Projections. Available at 10.5281/zenodo.6382554.

[r28] Goldstein EB, Coco G and Plant NG (2019) A review of machine learning applications to coastal sediment transport and morphodynamics. Earth-Science Reviews 194, 97–108. 10.1016/j.earscirev.2019.04.022.

[r29] Gomes da Silva P, Coco G, Garnier R and Klein AHF (2020) On the prediction of runup, setup and swash on beaches. Earth-Science Reviews 204, 103148. 10.1016/j.earscirev.2020.103148.

[r30] Hague BS, McGregor S, Murphy BF, Reef R and Jones DA (2020) Sea level rise driving increasingly predictable coastal inundation in Sydney, Australia. Earths Future 8(9), e2020EF001607. 10.1029/2020EF001607.

[r31] Harley M (2017) Coastal Storm Definition. In Ciavola P and Coco G (eds), Coastal Storms: Processes and Impacts. Chichester: Wiley, pp. 1–21. 10.1002/9781118937099.ch1.

[r32] Harley MD, Turner IL, Kinsela MA, Middleton JH, Mumford PJ, Splinter KD, Phillips MS, Simmons JA, Hanslow DJ and Short AD (2017) Extreme coastal erosion enhanced by anomalous extratropical storm wave direction. Scientific Reports 7(1), 6033–6039. 10.1038/s41598-017-05792-1. 28729733 PMC5519658

[r33] Harley MD, Turner IL and Short AD (2015) New insights into embayed beach rotation: The importance of wave exposure and cross-shore processes. Journal of Geophysical Research Earth Surface 120(8), 1470–1484. 10.1002/2014JF003390.

[r34] Harley MD, Turner IL, Short AD and Ranasinghe R (2010) Interannual variability and controls of the Sydney wave climate. International Journal of Climatology 30(9), 1322–1335. 10.1002/joc.1962.

[r35] Harley MD, Turner IL, Short AD and Ranasinghe R (2011a) Assessment and integration of conventional, RTK-GPS and image-derived beach survey methods for daily to decadal coastal monitoring. Coastal Engineering 58(2), 194–205. 10.1016/j.coastaleng.2010.09.006.

[r36] Harley MD, Turner IL, Short AD and Ranasinghe R (2011b) A reevaluation of coastal embayment rotation: The dominance of cross-shore versus alongshore sediment transport processes, Collaroy-Narrabeen Beach, Southeast Australia. Journal of Geophysical Research - Earth Surface 116(F4). 10.1029/2011JF001989.

[r37] Harley MD, Turner IL, Splinter KD, Phillips MS and Simmons JA (2016) Beach response to Australian east coast lows: A comparison between the 2007 and 2015 events, Narrabeen-Collaroy Beach. Journal of Coastal Research 75(sp1), 388–392. 10.2112/SI75-078.1.

[r38] Hazelwood M (2009) Geomorphology Smartline Geopackage. Geoscience Australia. Dataset. Available at https://pid.geoscience.gov.au/dataset/ga/104160.Accessed 14 July 2026.

[r39] Hedges TS and Mase H (2004) Modified Hunt’s equation incorporating wave setup. Journal of Waterway, Port, Coastal, and Ocean Engineering 130(3), 109–113. 10.1061/(ASCE)0733-950X(2004)130:3(109).

[r40] Holman RA (1986) Extreme value statistics for wave run-up on a natural beach. Coastal Engineering 9(6), 527–544. 10.1016/0378-3839(86)90002-5.

[r41] Holman R and Haller MC (2013) Remote sensing of the nearshore. Annual Review of Marine Science 5(1), 95–113. 10.1146/annurev-marine-121211-172408. 22809186

[r42] Holman RA and Stanley J (2007) The history and technical capabilities of Argus. Coastal Engineering 54(6-7), 477–491. 10.1016/j.coastaleng.2007.01.003.

[r43] Hunt IA (1959) Design of Seawalls and Breakwaters. Journal of the Waterways and Harbors Division 85(3), 123–152. 10.1061/JWHEAU.0000129.

[r44] Ibaceta R and Harley MD (2024) Data-driven modelling of coastal storm erosion for real-time forecasting at a wave-dominated embayed beach. Coastal Engineering 193, 104596. 10.1016/j.coastaleng.2024.104596.

[r45] Ibaceta R, Harley MD, Turner IL and Splinter KD (2023) Interannual variability in dominant shoreline behaviour at an embayed beach. Geomorphology 433, 108706. 10.1016/j.geomorph.2023.108706.

[r46] IPCC (2023) *Ocean, Cryosphere and Sea Level Change* . In *Climate Change 2021 – The Physical Science Basis: Working Group I Contribution to the Sixth Assessment Report of the Intergovernmental Panel on Climate Change*. Cambridge: Cambridge University Press, pp. 1211–1362. 10.1017/9781009157896.011.

[r47] Itzkin M, Palmsten ML, Buckley ML, Birchler JJ and Torres-Garcia LM (2025) Developing a decision tree model to forecast runup and assess uncertainty in empirical formulations. Coastal Engineering 195, 104641. 10.1016/j.coastaleng.2024.104641.

[r48] Jones B and O’Neill BC (2016) Spatially explicit global population scenarios consistent with the shared socioeconomic pathways. Environmental Research Letters 11(8). 10.1088/1748-9326/11/8/084003.

[r49] Khojasteh D, Haghani M, Nicholls RJ, Moftakhari H, Sadat-Noori M, Mach KJ, Fagherazzi S, Vafeidis AT, Barbier E, Shamsipour A and Glamore W (2023) The evolving landscape of sea-level rise science from 1990 to 2021. Communications Earth & Environment 4(1), 257. 10.1038/s43247-023-00920-4.

[r50] Khojasteh D, Rao S, McSweeney S, Ibaceta R, Nicholls RJ, French J, Glamore W, Largier JL, Adams J, Hughes MG, Barry M, Power HE, du J, Tucker TA, Cienfuegos R, Catalan PA and Hanslow D (2025) Intermittent estuaries deserve global attention as vulnerable and vital ecosystems. Communications Earth & Environment 6(1), 443. 10.1038/s43247-025-02428-5.

[r51] Kim LN, Brodie KL, Cohn NT, Giddings SN and Merrifield M (2023) Observations of beach change and runup, and the performance of empirical runup parameterizations during large storm events. Coastal Engineering 184, 104357. 10.1016/j.coastaleng.2023.104357.

[r52] Kim T and Lee W-D (2024) Prediction of wave runup on beaches using interpretable machine learning. Ocean Engineering 297, 116918. 10.1016/j.oceaneng.2024.116918.

[r53] Kinsela MA, Morris BD, Ingleton TC, Doyle TB, Sutherland MD, Doszpot NE, Miller JJ, Holtznagel SF, Harley MD and Hanslow DJ (2024) Nearshore wave buoy data from southeastern Australia for coastal research and management. Sci Data 11(1), 190. 10.1038/s41597-023-02865-x. 38347013 PMC10861473

[r54] Kinsela MA, Morris BD, Linklater M and Hanslow DJ (2017) Second-pass assessment of potential exposure to shoreline change in new South Wales, Australia, using a sediment compartments framework. Journal of Marine Science and Engineering 5(4). 10.3390/jmse5040061.

[r55] Kopp RE, Garner GG, Hermans THJ, Jha S, Kumar P, Reedy A, Slangen ABA, Turilli M, Edwards TL, Gregory JM, Koubbe G, Levermann A, Merzky A, Nowicki S, Palmer MD and Smith C (2023) The framework for assessing changes to sea-level (FACTS) v1.0: A platform for characterizing parametric and structural uncertainty in future global, relative, and extreme sea-level change. Geoscientific Model Development 16(24), 7461–7489. 10.5194/gmd-16-7461-2023.

[r56] Kulp SA and Strauss BH (2019) New elevation data triple estimates of global vulnerability to sea-level rise and coastal flooding. Nature Communications 10(1), 4844. 10.1038/s41467-019-12808-z. PMC6820795 31664024

[r57] Leaman CK, Harley MD, Splinter KD, Thran MC, Kinsela MA and Turner IL (2021) A storm hazard matrix combining coastal flooding and beach erosion. Coastal Engineering 170, 104001. 10.1016/j.coastaleng.2021.104001.

[r58] Limber PW, Barnard PL, Vitousek S and Erikson LH (2018) A model Ensemble for Projecting Multidecadal Coastal Cliff Retreat during the 21st century. Journal of Geophysical Research - Earth Surface 123(7), 1566–1589. 10.1029/2017JF004401.

[r59] Linklater M, Morris BD and Hanslow DJ (2023) Classification of seabed landforms on continental and island shelves. Frontiers in Marine Science 10. 10.3389/fmars.2023.1258556.

[r60] Lobeto H, Semedo A, Lemos G, Dastgheib A, Menendez M, Ranasinghe R and Bidlot J-R (2024) Global coastal wave storminess. Scientific Reports 14(1), 3726. 10.1038/s41598-024-51420-0. 38355634 PMC10866887

[r61] Lord D and Kulmar M (2000) The 1974 storms revisited: 25 years experience in ocean wave measurement along the south-east Australian coast. In Proceedings of the 27th International Conference on Coastal Engineering. Coastal Engineering 2000, 559–572. 10.1061/40549(276)44.

[r62] Lyard FH, Allain DJ, Cancet M, Carrère L and Picot N (2021) FES2014 global ocean tide atlas: Design and performance. Ocean Science 17(3), 615–649. 10.5194/os-17-615-2021.

[r63] Ochi M (2005) Ocean Waves: The Stochastic Approach. Cambridge: Cambridge University Press. 10.1017/CBO9780511529559.

[r64] Martins K, Blenkinsopp CE, Power HE, Bruder B, Puleo JA and Bergsma EWJ (2017) High-resolution monitoring of wave transformation in the surf zone using a LiDAR scanner array. Coastal Engineering 128, 37–43. 10.1016/j.coastaleng.2017.07.007.

[r65] Matheen N, Harley MD, Turner IL, Splinter KD, Simmons JA and Thran MC (2021) Bathymetric data requirements for operational coastal erosion forecasting using xbeach. Journal of Marine Science and Engineering 9(10). 10.3390/jmse9101053.

[r66] Mather A, Stretch D and Garland G (2011) Predicting extreme wave run-up on natural beaches for coastal planning and management. Coastal Engineering Journal 53(2), 87–109. 10.1142/S0578563411002288.

[r67] McInnes KL and Hubbert GD (2001) The impact of eastern Australian cut-off lows on coastal sea levels. Meteorological Applications 8(2), 229–243. 10.1017/S1350482701002110.

[r68] Mentaschi L, Vousdoukas MI, Dosio A, Voukouvalas E and Feyen L (2017) Global changes of extreme coastal wave energy fluxes triggered by intensified teleconnection patterns. Geophysical Research Letters 44(5), 2416–2426. 10.1002/2016gl072488.

[r69] Meucci A, Young IR, Hemer M, Kirezci E and Ranasinghe R (2020) Projected 21st century changes in extreme wind-wave events. Science Advances 6(24), eaaz7295–eaaz7210. 10.1126/sciadv.aaz7295. 32577512 PMC7286683

[r70] Middleton JH, Cooke CG, Kearney ET, Mumford PJ, Mole MA, Nippard GJ, Rizos C, Splinter KD and Turner IL (2013) Resolution and accuracy of an airborne scanning laser system for beach surveys. Journal of Atmospheric and Oceanic Technology 30(10), 2452–2464. 10.1175/JTECH-D-12-00174.1.

[r71] Morim J, Hemer M, Wang XL, Cartwright N, Trenham C, Semedo A, Young I, Bricheno L, Camus P, Casas-Prat M, Erikson L, Mentaschi L, Mori N, Shimura T, Timmermans B, Aarnes O, Breivik Ø, Behrens A, Dobrynin M, Menendez M, Staneva J, Wehner M, Wolf J, Kamranzad B, Webb A, Stopa J and Andutta F (2019) Robustness and uncertainties in global multivariate wind-wave climate projections. Nature Climate Change 9(9), 711–718. 10.1038/s41558-019-0542-5.

[r72] Morim J, Vitousek S, Hemer M, Reguero B, Erikson L, Casas-Prat M, Wang XL, Semedo A, Mori N, Shimura T, Mentaschi L and Timmermans B (2021) Global-scale changes to extreme ocean wave events due to anthropogenic warming. Environmental Research Letters 16(7). 10.1088/1748-9326/ac1013.

[r73] Morim J, Wahl T, Vitousek S, Santamaria-Aguilar S, Young I and Hemer M (2023) Understanding uncertainties in contemporary and future extreme wave events for broad-scale impact and adaptation planning. Earth’s Future 11(6), e2022EF003186. 10.1126/sciadv.ade3170 PMC9833663 36630499

[r74] Mortlock TR, Goodwin ID, McAneney JK and Roche K (2017) The June 2016 Australian East Coast Low: Importance of wave direction for coastal erosion assessment. Water 9. 10.3390/w9020121.

[r75] Muis S, Aerts JCJH, Antolínez Á, Dullaart JA, Duong JC, Erikson TM, Haarsma L, Apecechea RJ, Mengel MI, Le Bars M, O’Neill D, Ranasinghe A, Roberts R, Verlaan MJ, Ward M and Yan PJ (2023) Global projections of storm surges using high-resolution CMIP6 climate models. Earths Future 11(9), e2023EF003479. 10.1029/2023EF003479.

[r76] Neumann B, Vafeidis AT, Zimmermann J and Nicholls RJ (2015) Future coastal population growth and exposure to sea-level rise and coastal flooding - a global assessment. PLoS One 10(3), e0118571. 10.1371/journal.pone.0118571. 25760037 PMC4367969

[r77] Nielsen P and Hanslow DJ (1991) Wave Runup Distributions on Natural Beaches. Journal of Coastal Research 7(4), 1139–1152.

[r78] Nyberg B and Howell JA (2016) Global distribution of modern shallow marine shorelines. Implications for exploration and reservoir analogue studies. Marine and Petroleum Geology 71, 83–104. 10.1016/j.marpetgeo.2015.11.025.

[r79] O’Grady JG, McInnes KL, Hemer MA, Hoeke RK, Stephenson AG and Colberg F (2019) Extreme water levels for Australian beaches using empirical equations for shoreline wave setup. Journal of Geophysical Research, Oceans 124(8), 5468–5484. 10.1029/2018JC014871.

[r80] Oliver TSN, Kinsela MA, Doyle TB and McLean RF (2024) Foredune erosion, overtopping and destruction in 2022 at Bengello Beach, southeastern Australia. Cambridge Prisms: Coastal Futures 2, e7. 10.1017/cft.2024.8. 40881329 PMC12337608

[r81] Palmsten ML and Holman RA (2012) Laboratory investigation of dune erosion using stereo video. Coastal Engineering 60, 123–135. 10.1016/j.coastaleng.2011.09.003.

[r82] Phillips MS, Blenkinsopp CE, Splinter KD, Harley MD and Turner IL (2019) Modes of berm and Beachface recovery following storm reset: Observations using a continuously scanning Lidar. Journal of Geophysical Research - Earth Surface 124(3), 720–736. 10.1029/2018JF004895.

[r83] Portch CE, Cuttler MVW, Buckley ML, Hansen JE and Lowe RJ (2023) Wave runup and inundation dynamics on a perched beach. Geomorphology 435, 108751. 10.1016/j.geomorph.2023.108751.

[r84] Power HE, Gharabaghi B, Bonakdari H, Robertson B, Atkinson AL and Baldock TE (2019) Prediction of wave runup on beaches using gene-expression programming and empirical relationships. Coastal Engineering 144, 47–61. 10.1016/j.coastaleng.2018.10.006.

[r85] Pronk M, Hooijer A, Eilander D, Haag A, de Jong T, Vousdoukas M, Vernimmen R, Ledoux H and Eleveld M (2024) DeltaDTM: A global coastal digital terrain model. Sci Data 11(1), 273. 10.1038/s41597-024-03091-9. 38448476 PMC10917791

[r86] Reguero BG, Losada IJ and Méndez FJ (2019) A recent increase in global wave power as a consequence of oceanic warming. Nature Communications 10(1), 205–214. 10.1038/s41467-018-08066-0. PMC6331560 30643133

[r87] Roelvink D, Reniers A, van Dongeren A, van Thiel de Vries J, McCall R and Lescinski J (2009) Modelling storm impacts on beaches, dunes and barrier islands. Coastal Engineering 56(11-12), 1133–1152. 10.1016/j.coastaleng.2009.08.006.

[r88] Ruggiero P, Komar PD, Mcdougal WG, Marra JJ and Beach RA (2001) Wave Runup, Extreme Water Levels and the Erosion of Properties Backing Beaches. Journal of Coastal Research 17(2), 407–419.

[r89] Saye SE, van der Wal D, Pye K and Blott SJ (2005) Beach–dune morphological relationships and erosion/accretion: An investigation at five sites in England and Wales using LIDAR data. Geomorphology 72(1-4), 128–155. 10.1016/j.geomorph.2005.05.007.

[r90] Shand TD, Goodwin ID, Carley JT, Browning S, Coghlan IR and Peirson WL (2011b) NSW Coastal Inundation Hazard Study: Coastal Storms and Extreme Waves.

[r91] Shand RD, Shand TD, Mccomb PJ and Johnson DL (2011) Evaluation of Empirical Predictors of Extreme Run-Up Using Field Data. https://api.semanticscholar.org/CorpusID:62882690

[r92] Shope JB, Erikson LH, Barnard PL, Storlazzi CD, Serafin K, Doran K, Stockdon H, Reguero B, Mendez F, Castanedo S, Cid A, Cagigal L and Ruggiero P (2022) Characterizing storm-induced coastal change hazards along the United States west coast. Scientific Data 9(1), 224. 10.1038/s41597-022-01313-6. 35606384 PMC9126972

[r93] Short AD (2007) Beaches of the New South Wales Coast: A Guide to their Nature, Characteristics, Surf and Safety. Sydney: Sydney University Press. 10.2307/jj.4688116.

[r94] Short AD and Trenaman NL (1992) Wave climate of the Sydney region, an energetic and highly variable ocean wave regime. Marine and Freshwater Research 43(4), 765–791. 10.1071/MF9920765.

[r95] Simmons JA, Splinter KD, Harley MD and Turner IL (2019) Calibration data requirements for modelling subaerial beach storm erosion. Coastal Engineering 152, 103507. 10.1016/j.coastaleng.2019.103507.

[r96] Splinter KD, Harley MD and Turner IL (2018) Remote sensing is changing our view of the coast: Insights from 40 years of monitoring at Narrabeen-Collaroy, Australia. Remote Sensing 10. 10.3390/rs10111744.

[r97] Stockdon HF, Doran KS and Sallenger AH (2009) Extraction of lidar-based dune-crest elevations for use in examining the vulnerability of beaches to inundation during hurricanes. Journal of Coastal Research 10053, 59–65. 10.2112/SI53-007.1.

[r98] Stockdon HF, Holman RA, Howd PA and Sallenger AH (2006) Empirical parameterization of setup, swash, and runup. Coastal Engineering 53(7), 573–588. 10.1016/j.coastaleng.2005.12.005.

[r99] Stockdon HF, Holman RA and Survey USG (2011). Observations of Wave Runup, Setup, and Swash on Natural Beaches, Data Series. Reston, VA. Available at 10.3133/ds602.

[r100] Stockdon HF, Long JW, Palmsten ML, Van der Westhuysen A, Doran KS and Snell RJ (2023) Operational forecasts of wave-driven water levels and coastal hazards for US gulf and Atlantic coasts. Communications Earth & Environment 4(1), 169. 10.1038/s43247-023-00817-2.

[r101] Stockdon HF, Thompson DM, Plant NG and Long JW (2014) Evaluation of wave runup predictions from numerical and parametric models. Coastal Engineering 92, 1–11. 10.1016/j.coastaleng.2014.06.004.

[r102] Suanez S, Cancouët R, Floc’h F, Blaise E, Ardhuin F, Filipot JF, Cariolet JM and Delacourt C (2015) Observations and predictions of wave runup, extreme water levels, and medium-term dune erosion during storm conditions. Journal of Marine Science and Engineering 3(3), 674–698. 10.3390/jmse3030674.

[r103] Taherkhani M, Vitousek S, Barnard PL, Frazer N, Anderson TR and Fletcher CH (2020) Sea-level rise exponentially increases coastal flood frequency. Scientific Reports 10(1), 6466. 10.1038/s41598-020-62188-4. 32300112 PMC7162943

[r104] Thom B (2022) Coastal management and the Australian government: A personal perspective. Ocean and Coastal Management 223, 106098. 10.1016/j.ocecoaman.2022.106098.

[r105] Toimil A, Losada IJ, Álvarez-Cuesta M and Le Cozannet G (2023) Demonstrating the value of beaches for adaptation to future coastal flood risk. Nature Communications 14(1), 3474. 10.1038/s41467-023-39168-z. PMC10261140 37308502

[r106] Toimil A, Losada IJ, Nicholls RJ, Dalrymple RA and Stive MJF (2020) Addressing the challenges of climate change risks and adaptation in coastal areas: A review. Coastal Engineering 156, 103611. 10.1016/j.coastaleng.2019.103611.

[r107] Tolman HL (2014) User Manual and System Documentation of WAVEWATCH III R Version 4.18. https://polar.ncep.noaa.gov/waves/wavewatch/manual.v4.18.pdf

[r108] Viola CNA, Verdon-Kidd DC, Hanslow DJ, Maddox S and Power HE (2021) Long-Term Dataset of Tidal Residuals in New South Wales, Australia. Data 6. 10.3390/data6100101.

[r109] Viola CNA, Verdon-Kidd DC and Power HE (2024) Spatially varying impacts of pacific and southern ocean climate modes on tidal residuals in New South Wales, Australia. Estuarine, Coastal and Shelf Science 305, 108869. 10.1016/j.ecss.2024.108869.

[r110] Vitousek S, Barnard PL, Fletcher CH, Frazer N, Erikson L and Storlazzi CD (2017) Doubling of coastal flooding frequency within decades due to sea-level rise. Scientific Reports 7(1), 1399–1399. 10.1038/s41598-017-01362-7. 28522843 PMC5437046

[r111] Vitousek S, Buscombe D, Vos K, Barnard PL, Ritchie AC and Warrick JA (2023) The future of coastal monitoring through satellite remote sensing. Cambridge Prisms: Coastal Futures 1, e10. 10.1017/cft.2022.4.

[r112] Vitousek S, Cagigal L, Montaño J, Rueda A, Mendez F, Coco G and Barnard PL (2021) The application of ensemble wave forcing to quantify uncertainty of shoreline change predictions. Journal of Geophysical Research - Earth Surface 126(7). 10.1029/2019jf005506.

[r113] Vos K, Deng W, Harley MD, Turner IL and Splinter KDM (2022) Beach-face slope dataset for Australia. Earth System Science Data 14(3), 1345–1357. 10.5194/essd-14-1345-2022.

[r114] Vos K, Splinter KD, Harley MD, Simmons JA and Turner IL (2019) CoastSat: A Google earth engine-enabled Python toolkit to extract shorelines from publicly available satellite imagery. Environmental Modelling & Software 122, 104528. 10.1016/j.envsoft.2019.104528.

[r115] Vos K, Harley MD, Splinter KD, Walker A and Turner IL (2020) Beach slopes from satellite-derived shorelines. Geophysical Research Letters 47(14). 10.1029/2020GL088365.

[r116] Vos K, Harley MD, Turner IL and Splinter KD (2023) Pacific shoreline erosion and accretion patterns controlled by El Niño/southern oscillation. Nature Geoscience 16(2), 140–146. 10.1038/s41561-022-01117-8.

[r117] Vousdoukas MI, Wziatek D and Almeida LP (2012) Coastal vulnerability assessment based on video wave run-up observations at a mesotidal, steep-sloped beach. Ocean Dynamics 62(1), 123–137. 10.1007/s10236-011-0480-x.

[r118] Vousdoukas MI, Mentaschi L, Voukouvalas E, Verlaan M, Jevrejeva S, Jackson LP and Feyen L (2018) Global probabilistic projections of extreme sea levels show intensification of coastal flood hazard. Nature Communications 9(1), 2360. 10.1038/s41467-018-04692-w. PMC6006144 29915265

[r119] Wahl T, Haigh ID, Nicholls RJ, Arns A, Dangendorf S, Hinkel J and Slangen AB (2017) Understanding extreme sea levels for broad-scale coastal impact and adaptation analysis. Nature Communications 8(1), 16075. 10.1038/ncomms16075. PMC5504349 28685752

[r120] Wright LD and Short AD (1984) Morphodynamic variability of surf zones and beaches: A synthesis. Marine Geology 56(1-4), 93–118. 10.1016/0025-3227(84)90008-2.

[r121] Wright LD, Short AD and Green MO (1985) Short-term changes in the morphodynamic states of beaches and surf zones: An empirical predictive model. Marine Geology 62(3-4), 339–364. 10.1016/0025-3227(85)90123-9.

[r122] Zornoza-Aguado M, Pérez-Díaz B, Cagigal L, Castanedo S and Méndez FJ (2024) An efficient metamodel to downscale total water level in open beaches. Estuarine, Coastal and Shelf Science 299, 108705. 10.1016/B978-0-08-102927-5.00028-X.

