# Supplementary material for: Challenges and opportunities in predicting coastal total water levels from empirical runup models: Insights from major storms in Southeast Australia
Source: Camb Prism Coast Futur. 2026 Jun 3;4:e21. doi: 10.1017/cft.2026.10043 (PMC13458094; doi:10.1017/cft.2026.10043)
Supplement: Supplementary file 1 [file S2754720526100432sup001.docx]

**Challenges and opportunities in predicting coastal total water levels from empirical runup models: Insights from major storms in Southeast Australia**

Raimundo Ibaceta et al.

# **Appendix A: Relationship between observed *TWL debris lines, associated* with *R_max_* and *R_2%_* and *SWL***

Marine debris observations hypothetically correspond to *TWL* modelling obtained from the maximum combined levels of still water levels (*SWL*) and maximum runup (*R_max_)* during a storm, i.e., *TWL_observed_ = (SWL + R_max_*)*_max,_* This assumes that the debris lines were deposited not at the maximum still water level (*SWL*) or at the maximum runup (*R_max_*) individually, but at the maximum combined elevation of both during the storm.

However most runup formulae provide values of *R_2%,_* not *R_max_*. Here we derive a scaling factor *α* that relates *R_max_*, the maximum runup contribution (excluding *SWL*) during a storm and *R_2%_*, which is the runup height exceeded by two percent of waves (*R_2%_ = αR_max_)*.

Assuming that the energy spectrum of the instantaneous shoreline elevation time series is narrow-banded, the probability distribution of the maxima, i.e., wave runup heights *R*, should approximate a Rayleigh distribution (Cartwright and Longuet-Higgins, 1956). The Rayleigh probability density function for *R* can be written as:

$p\left( R;\sigma\right)=\frac{2R}{\sigma^{2}}e^{\left( {-R^{2}}/{\sigma^{2}} \right)}$	(A1)

where *p* is probability density and *σ* is the scale parameter (or spread) of the distribution (i.e., vertical runup scale). The cumulative distribution function $P\left( R;\sigma\right)$ is obtained through integration and can be expressed as:

$P\left( R;\sigma\right)=\int_{0}^{R} p\left( R;\sigma\right).dR=1-e^{\left( {-R^{2}}/{\sigma^{2}} \right)}$	(A2)

The two percent exceedance runup height (*R_2%_*) is calculated from the cumulative distribution function as below:

$P\left( R;\sigma\right)=1-e^{\left( {-R^{2}}/{\sigma^{2}} \right)}=0.98$	(A3)

which yields:

$R_{2\%}=\sqrt{-\sigma^{2}ln(0.02)}$	(A4)

It is not possible to obtain the zero percent exceedance value (i.e., *R_max_*) in the same way since the $ln(0)$ is infinite. To address this, it is possible to employ a different approach by defining *R_max_* as the extreme value, which is the largest value of a random variable expected to occur within a specified number of observations *n*, and is estimated using the principles of order statistics. Using this approach the general form of the probability density function for the extreme value is related to both the probability density function and cumulative distribution function of the initial set of observations. This relationship in our notation is given by Ochi (2005):

$p\left( R_{max} \right)=n\left[ p\left( R_{max} \right)P\left( R_{max} \right)^{n-1} \right]$	(A5)

For an initial probability density function that follows a Rayleigh distribution, Ochi (2005) showed that the shape of *p(R_max_)* is sharply concentrated around its modal value, and it is, therefore, reasonable to assume this mode as the most probable extreme value. This probable extreme value can be obtained by finding the maximum of *p(R_max_)*, thus by solving:

$\frac{d}{dR_{max}}p\left( R_{max} \right)=0$	(A6)

which yields

$p^{'}(R_{max};\sigma)P(R_{max};\sigma)+\left( n-1 \right)[{p(R_{max};\sigma)]}^{2}=0$	(A7)

Substituting (A1) and (A2) into (A7) it can be shown that

$R_{max}=\sigma\sqrt{ln(n)}$	(A8)

We now have an expression for *R_2%_* and *R_max_* that are both linearly related to σ. Solving Eq. A8 for σ and substituting into Eq. A4 yield and expression for *R_2%_* that is independent of σ:.

$R_{2\%}=R_{max}\sqrt{-\frac{1}{\ln\left( n \right)}ln(0.02)}$	(A9)

Thus the scaling factor *α* to convert *R_max_* to *R_2%_* is

$\alpha=\sqrt{-\frac{1}{\ln\left( n \right)}ln(0.02)}$	(A10)

Equation A9 offers a method to convert the measured marine debris line elevation corresponding to *R_max_* (i.e., *TWL_observed_*) into an estimate of *R_2%_* (*R_2%_ = αR_max_)* without complications related to estimating *L*, or vice versa:

*αTWL_observed_ = (αSWL + R_2%_)_max_*	(A11)

This conversion is practical, but it requires selecting an appropriate value for *n* in Equation A10. Note that as *n* increases, the likelihood of observing a larger *R_max_* also increases. Assuming that the debris line formation likely coincides with the time when the water level is largely stationary for an hour around the peak of the storm, a suitable and pragmatic choice for *n* would be the number of waves occurring in one hour. In New South Wales, this can be easily estimated using the regional long-term average wave period *T ~ 10 s,* equivalent to *n* ~ 360 waves per hour. This value yields a scaling factor of *α =* 0.81*.* Considering other range of wave periods typical in NSW (e.g., 7 to 15s) results in negligible differences in *α* (within 4%).

# **Appendix B**

Figure A- 1. Example scatter plots of total water level modelling (TWL = SWL + R, 471 observations) for three different beach slope specifications (represented by different colours from left to right) and three runup formulae (displayed from top to bottom panels). Dots indicate individual obervations and their corresponding modelled value. The black dashed lines indicate a perfect fit (1:1). Coloured solid lines are linear fits obtained from the modelled data and are included to facilitate the interpretation of the model’s skill and performance.
