# Supplementary material for: Challenges and opportunities in predicting coastal total water levels from empirical runup models: Insights from major storms in Southeast Australia
Source: Camb Prism Coast Futur. 2026 Jun 3;4:e21. doi: 10.1017/cft.2026.10043 (PMC13458094; doi:10.1017/cft.2026.10043)
Supplement: Peer review history

# Reviewer report: Review: Challenges and opportunities in predicting coastal total water levels from empirical runup models: Insights from major storms in Southeast Australia — R0/PR2

**DOI:** 10.1017/cft.2026.10043.pr2

**Authors:** Tom Baldock

**Affiliations:** University of Queensland, Australia

There seems to be no option to upload an annotated file from working offline.

Please email me if you need it to follow the comments.

File: CFT-2025-0044_Proof_hi TB.pdf

Annotation summary:

--- Page 5 ---

Highlight (color #FFC100), Tom Baldock:

Also occurs elsewhere - Tomas Buezen, should be Buezen.

Highlight (color #9643FC), Tom Baldock:

This definition of SWL, while I understand the traditional meaning, does not make much sense when talking about tidal and non-tidal residuals being “still” water. It is really the time-averaged water level (over some chosen instrument time interval) well outside the surf zone, unless those tide gauges become inside the surf zone. Perhaps you can propose a sensible definition for future use.

--- Page 7 ---

Highlight (color #9643FC), Tom Baldock:

yes, but a very large majority of the Stockdon data were from one beach I think.

--- Page 25 ---

Highlight (color #FFC100), Tom Baldock:

Tomas

--- Page 29 ---

Highlight (color #9643FC), Tom Baldock:

units missing

Highlight (color #FFC100), Tom Baldock: line 696

RMSE = 0.6, units missing

Highlight (color #9643FC), Tom Baldock:

This implies the surf beat term in Stockdon is inaccurate for these data and that a surfbeat amplitude linearly proportional to Ho (as in H86 and A17) is better at representing that influence. This is consistent with surf beat by breakpoint generated long waves as described in Baldock and Huntley (2002), section 4d.

--- Page 30 ---

Highlight (color #FFC100), Tom Baldock:

Rmax,,

--- Page 31 ---

Highlight (color #FFC100), Tom Baldock:

Ochi (

762 2005)

Highlight (color #9643FC), Tom Baldock: (A8)

Equivalent to the standard expression for the highest of N waves, with Hrms=L.

--- Page 32 ---

Highlight (color #9643FC), Tom Baldock: (A9)

Alpha should be on R2%

Highlight (color #9643FC), Tom Baldock:

Did you try Nielsen (2009) section 2.4.5?

# Reviewer report: Review: Challenges and opportunities in predicting coastal total water levels from empirical runup models: Insights from major storms in Southeast Australia — R0/PR3

**DOI:** 10.1017/cft.2026.10043.pr3

**Authors:** Katherine Serafin

**Affiliations:** Geography, University of Florida, United States

<b>Summary:</b>

The manuscript “Challenge and opportunities in predicting coastal water levels from empirical runup models: Insights from major storms in Southeast Australia” uses estimates of maximum total water levels (TWLs) from RTK-GPS surveyed wrack lines on beaches after a set of storms to show there is variability in accuracy of TWL estimates when using different runup formulations. A comparison of 7 different commonly used runup models show varied performance and that 2 models perform the best – one which was created based on data local to the area and another developed 30 years earlier at a field site in Duck, North Carolina. The need to address TWL uncertainties is discussed in the context of the work related to broader challenges and uncertainties in modeling TWLs. This is a timely and valuable study that addresses critical aspects of total water levels, particularly given the scarcity of extreme TWL observations and the limited availability of comparisons with modeled TWLs. It is well written and organized. I believe this manuscript has the potential to make a valuable contribution to the literature. However, there are some points that need more discussion, including uncertainties generated from other parameters impacting TWL modeling beyond wave runup. Below, I outline several comments that, if addressed, could further enhance its quality.

<b>General Comments:</b>

1. The underlying assumption of the relationship between the proxy for the maximum storm-driven TWL, the wrack line, and wave runup, is that the wrack line is formed during the maximum wave runup event (Rmax). Wrack line measurements were taken the day following the peak in wave height. Because TWL = SWL + wave runup, how valid is this assumption? An additional panel in Figure 3c showing the TWL from one or a few R2% formulations – or even a new figure showing the R2% and TWL -- would be helpful to explain this assumption. Because of the differences in wave runup formulations, slight differences in wave height and period could lead to the SWL having a higher contribution to the TWL, dictating the timing of the maximum TWL, which would drive the location of the wrack line. It would be interesting to see whether the peak TWL of the event always aligns with the peak Hs and/or R2% as the magnitude of the TWL could be very different as the R2% combines with SWL. The influence of the SWL to the TWL is important for understanding the wrack line’s relationship to the wave runup formulas which is the main component of the TWL discussed within.

2. The results primarily evaluate the accuracy of runup formulations in representing TWL, but it is important to note that SWL is also a critical component of peak TWL magnitude. Likewise, the accuracy of wave parameters from the wave model could plays a significant role in spatial variability. It would be helpful to consider whether variation in coefficient of determination or RMSE across modeling choices might partly reflect these additional factors, rather than limitations of the runup formulations alone. For instance, slight differences in wave height and/or transformation (if modeled waves in the nearshore don’t match well with nearshore waves) could introduce nonlinear differences in R2% across formulations. Wave period, which is often less well validated than wave height but central to many runup formulations, could also contribute. Similarly, uncertainties in modeled wave values, or mismatches in the magnitude or timing of SWL relative to the location of interest (e.g., when tide gauge data are used at some distance from study beaches, I’m not entirely clear how SWL is considered), could propagate into the runup results. As an example, the Stockdon formulation for dissipative beaches may perform differently depending on specific input wave characteristics, highlighting the importance of considering these other sources of variability.

When uncertainties in modeled wave characteristics are combined with potential uncertainties in SWLs (e.g., how well does a tide gauge represent a SWL that is not close to the gauge?), then the predicted timing and magnitude of the TWL peak could be different, leading to differences in comparisons to the observed TWL. While I don’t doubt the formulation is a larger driver of uncertainty, it would be good/helpful to see additional transparent discussion on other drivers of the uncertainty in TWL (modeled SWL, modeled Waves, nearshore bar morphology (e.g., Sherwood et al., 2023; Cohn et al., 2016) which are also important for TWL modeling and not discussed at all.

Sherwood, C. R., Aretxabaleta, A. L., Traykovski, P., Over, J. S. R., Lyons, E., Foster, D. S., ... & Sogut, E. (2023). Contributions to uncertainty in runup forecasts. In Coastal Sediments 2023: The Proceedings of the Coastal Sediments 2023 (pp. 401-418).

Cohn, N., & Ruggiero, P. (2016). The influence of seasonal to interannual nearshore profile variability on extreme water levels: Modeling wave runup on dissipative beaches. Coastal Engineering, 115, 79-92.

3. The term “foreshore beach slope” is often used throughout the wave runup literature, to describe the slope around the intertidal area/or where wave runup is operating. This term is used in line 391 to describe the CoastSat-derived slopes, and has the same definition as the slope of the partial intertidal zone (MSL to MHWS). Is there a reason the term foreshore slope is not used to explain the first method defining the beach slope? Another commonly used slope metric is the mean beach slope, taken as the beach slope from the shoreline to the dune toe (e.g., Stockdon et al., 2012; Stockdon et al., 2023) since the waves act over the entire beach during extreme conditions. How would this value relate to the MSL to berm slope?

Stockdon, H. F., Doran, K. S., Thompson, D. M., Sopkin, K., L., Plant, N. G., & Sallenger, A. H. (2012). National Assessment of Hurricane-Induced Coastal Erosion Hazards: Gulf of Mexico (Open-File Report) [Open-File Report]. USGS.

4. The discussion highlights the results from this manuscript that the best choice for beach slope is the MSL to the Berm, rather than the foreshore slope (MSL to MHWS). However, the results should be more clearly described as being based on maximum/storm values, which have the potential to act moreover the beach face (e.g., the justification Stockdon et al., 2012 uses for using the mean beach slope), compared to daily or hourly TWLs. The word choice matters a bit because there might be a difference for when the foreshore slope can be used to provide better results versus another beach slope, so tailoring the discussion a bit to extreme TWLs is important. Also, how do the CoastSat and lidar foreshore slopes compare?

5. The study emphasizes the performance of the Atkinson et al., 2017 model in reproducing observations and concludes that site-specific data are essential for accurately modeling runup. However, the strong performance of Holman, 1986 appears to challenge this conclusion! While there is a discussion that they have similar forms, the Holman model did come 30 years before the Atkinson model, based only on Duck, North Carolina data. I think this needs broader discussion – about the reasons why this model might do so well here, because the answer isn’t only runup model need to be formulated using data the specific beach of interest. Model accuracy may also be strongly tied to the similarity between the calibration dataset and the conditions at the study site (Itzkin et al., 2025 has a nice figure). Perhaps introducing a wide range of non-representative conditions can compromise performance, as constants and coefficients are then optimized for a less relevant fit

6. Critiques about machine learning (ML) models developed to estimate R2% are discussed, noting that they may be overly generalized and prone to significant errors when applied beyond their training range. This is true but is not the only way to develop ML models. New work from Itzkin et al., 2025 builds a decision tree model for forecasting R2% and TWLs that uses a weighted ensemble where the runup models being used can change over time with hydrodynamic conditions and the beach state so that the most appropriate formulations are always used. One of the benefits of this approach is that it realistically weighs runup models as it runs without requiring user input or prior knowledge of the system. It still isn’t as accurate as it could be (and is modeling/comparing hourly runup, rather than just extreme events) but provides an interesting step in the utility of R2 modeling and ML to ensure appropriate use case of the models based on the morphological/hydrodynamic conditions when the model was developed, rather than some a priori assumption.

Itzkin, M., Palmsten, M. L., Buckley, M. L., Birchler, J. J., & Torres-Garcia, L. M. (2025). Developing a decision tree model to forecast runup and assess uncertainty in empirical formulations. Coastal Engineering, 195, 104641.

7. The section beginning on line 612 is interesting to show why incorporating uncertainty in beach slope is important. Some of the way it’s written suggests to me that no one has thought about this before, however, Stockdon et al., 2023 (which is cited in the paper) is already leveraging the incorporation of beach slope uncertainty into forecasts and probabilities of coastal change (e.g., Stockdon et al., 2013) based on the Doran et al., 2015 method, and have been for years now. The sentence “ This assessment demonstrates the potential to leverage the uncertainty of this key input variable (β) as a source of uncertainty for TWL modelling” seems like a good place to note the studies/models that are presently incorporating these ideas into TWL modelling.

Stockdon, H. F., Doran, K. S., Thompson, D. M., Sopkin, K. L., & Plant, N. G. (2013). National assessment of hurricane-induced coastal erosion hazards: Southeast Atlantic Coast (No. 2013-1130). US Geological Survey.

8. TWL errors in the discussion section 4.2.2 should probably be compared to SLR uncertainties, not median/likely projections of SLR, especially as you get out to 2100, the uncertainties of the SLR will increase. These comparisons are more relatable to each other.

9. It would be helpful to know the average beach slopes across the different locations being studied/evaluated.

<b>Line by Line Comments:</b>

Line 269 – 270: Were measurements taken/were there any instances when the wrack line occurred interacted with vegetation (e.g., dune grass) or interacted/overtopped the dune? Or were all the wrack lines surveyed located on the beachface? This additional detail would be helpful to know. I ask because I’m wondering about there are any assumptions with the location of the wrack line and its relationship to the TWL, like the potential for it to be snagged on grasses (e.g., Bush et al., 1996).

Bush, D. M., Young, R. S., Webb, C. A., & Thieler, E. R. (1996). Soundside impacts of a northward tracking tropical cyclone: Hurricane Emily (31Aug93), Cape Hatteras area, North Carolina. Journal of Coastal Research, 229-239.

Lines 286-287: A figure showing where the transects are, at least for some locations, and how many of the TWL observations are at each one would be helpful. For example, are the 345 transects distributed evenly across the coastal stretch shown in Figure 3? This could be located in the appendix if not room in the main paper,

Lines 333 – 337: I’m interested in what this tool is, as “tool” is a pretty generic description. Are they lookup tables? Wave transformation functions? Surrogate/meta models? How many joint conditions (H,T,D) were utilized? Is the procedure an interpolation (e.g., Serafin et al., 2019) or nonlinear interpolation (e.g., Camus et al., 2011)? How recent are the DEMs? This may be in some of the previous papers but it’s worthwhile mentioning some of the details here.

Similarly, I am assuming the offshore waves inputted into the model for Newcastle and all locations N of Sydney area use waves from the offshore buoy off Sydney. How representative are these wave directions and other characteristics to Newcastle?

Camus, P., Mendez, F. J., & Medina, R. (2011). A hybrid efficient method to downscale wave climate to coastal areas. Coastal Engineering, 58(9), 851-862.

Serafin, K. A., Ruggiero, P., Barnard, P. L., & Stockdon, H. F. (2019). The influence of shelf bathymetry and beach topography on extreme total water levels: Linking large-scale changes of the wave climate to local coastal hazards. Coastal Engineering, 150, 1-17.

Line 531 – 534: Wouldn’t this line be the same for the Holman model, which does about equally (statistically) well?

Line 565: I get the idea of the sentence “success of these methods will only be achieved by continuous data collection efforts during storms” as we need more real time measurements during storms, but what if that can’t be done? It might be unrealistic broadly). Also Line 172 – 174 that says pre-storm slope estimates can be accurate compared to estimates during morphological change so can this be rephrased to be more specific?

Line 581: The statement suggesting Lidar derived slopes are better suited for coastal risk assessments than satellite derived slops should be rephrased because it’s not necessarily the fact that the slope is satellite derived but the way it’s derived – the MSL-MHWS/foreshore slope metric. It can also be calculated from Lidar, which does not do as well as the MSL to Berm measurement. So I’d highlight the type of slope rather than the metric of developing it here.

Line 601: Should this be considered R2% modeling instead of TWL modeling? Because there is no variation in the SWL forcing, it seems that only the variation in the R2% is being tested.

Line 608: Beuzen citation odd to include first name.

Lines starting 639: 4.2.2 – this discussion could (should?) also highlight Wahl et al. 2017 work that show uncertainties in extreme still water levels (Surge + tide, more ore less) are greater than uncertainties in SLR.

Wahl, T., Haigh, I. D., Nicholls, R. J., Arns, A., Dangendorf, S., Hinkel, J., & Slangen, A. B. (2017). Understanding extreme sea levels for broad-scale coastal impact and adaptation analysis. Nature communications, 8(1), 16075.

Line 687: Should this be considered R2% modeling? Because there is no variation in the SWL forcing, it seems that only the variation in the R2% is being tested.

Line 742: Why is the scale parameter represented as L instead of σ, as commonly expressed in this formulation? Substituting R for x makes sense, since x = wave runup. However, defining the scale parameter as L is a bit confusing since L is defined as the wave length in wave runup formulations. I also find the description of what L is to be confusing, using scale (I think) in two different ways – “… and L is the scale parameter of the distribution (i.e. vertical runup scale).” I better way to describe the scale parameter is the spread or width of the distribution.

Line 787-789: How variable is the result with a different n? Why not use the wave period during the peak wave event, rather than a long term average to make it vary storm by storm?

References: Baird publication link is incorrect and does not start with https://doi.org, as far as I can tell.

Figures and Tables

Table 1: I am glad you decided against providing the Power et al., 2019 full formulation within the table!

Figure 2: what beach slope was used in this calculation?

Figure 4: if there are no bars does that mean there was no debris surveyed? If yes, is it because there was a backing wall or because there was no extreme runup event to leave a wrack line? If a wall/cliff, can that be added to the figure?

# Editor report: Recommendation: Challenges and opportunities in predicting coastal total water levels from empirical runup models: Insights from major storms in Southeast Australia — R0/PR4

**DOI:** 10.1017/cft.2026.10043.pr4

**Affiliations:** Faculty of Engineering, UBC Institute for the Oceans and Fisheries, Canada

I have added my comments as Handling Editor, and agree with the recommendation of major revisions.

# Editor report: Decision: Challenges and opportunities in predicting coastal total water levels from empirical runup models: Insights from major storms in Southeast Australia — R0/PR5

**DOI:** 10.1017/cft.2026.10043.pr5

**Affiliations:** Faculty of Engineering, UBC Institute for the Oceans and Fisheries, Canada

No accompanying comment.

# Reviewer report: Review: Challenges and opportunities in predicting coastal total water levels from empirical runup models: Insights from major storms in Southeast Australia — R1/PR7

**DOI:** 10.1017/cft.2026.10043.pr7

**Authors:** Katherine Serafin

**Affiliations:** Geography, University of Florida, United States

This is the second time I’ve reviewed this manuscript. It was already a strong manuscript, just needed some clarifications, and I believe it has improved with additional context added. Again, I believe this is a valuable study considering the limited availability of observations of TWLs at the coast.

A few minor remaining suggestions/comments

• I appreciate the addition of a line explaining the uncertainty arising from data inputs in the manuscript as a response to one of my previous comments. But I think where it is located is a little out of place because by line 235 there has been no discussion of inputs – this might be better aligned in the discussion section, where this limitation/point has also been highlighted.

• It feels like the results of Holman are hidden in the appendix when they do almost (and probably equally as well, statistically) as Atkinson (figure 6). I understand showing only a handful of different runup formulations results in the manuscript and some in the appendix/supplemental info to highlight the difference between methods (Figure 6), but it I feel that the language around Atkinson (e.g., Line 519 “superior predictions”) are to sway the reader to the impressiveness of that result while not highlighting the equivalency of the Holman result. It would be great (and more transparent!) to also have some of the stats for Holman in the main manuscript, even if the figure of the results remains in the appendix. E.g., Line 519, what is the RMSE, BIAS p^2?. Adding this information in will also help to support paragraph Line 556 which discusses Atkinson as the most accurate model, if the performance metrics are indeed slightly better.

• It’s curious that in Figure 4, there are locations that had observations of marine debris in coastal stretches 2 and 3 during event #2 and #3 but not event #1. Any idea why this might be? Was the data at location #1 truncated in those locations so not included, or is there some site specific feature/increase in the TWL in these locations during those storms?

• I think the presentation of all of the figures are great and summarize the results well, but they also leave me to ask – do the TWL predictions do better in some locations/beaches over other ones? E.g., if you remade Figure 5 or 6 for individual coastal stretches, would you see more agreement in some locations over others? This isn’t something to do for this manuscript, but it is interesting to think about the influence of site specific variability.

Line-by-Line

Line 77: I’d consider changing the word “from” to “using” i.e., “…outperformed the models USING emerging satellite-derived slopes…” as the models being described are runup models and they use beach slope, they’re not from beach slopes.

Line 114: Should “shelfs” be “shelves”?

Figure 1: Mean sea level is missing from the conceptual diagram, unless it’s being considered in the tide component. It is in the definition of the SWL on line 122 (msl + tide+ NTR)

Line 160: cite Figure 1a but there is no “a” in figure 1

Lines 197, Table 1, 551, etc.: describe the “Duck Research Facility Centre” But that’s not its name. It’s usually called the FRF for the Field Research Facility (in Duck, NC) https://www.erdc.usace.army.mil/Media/Fact-Sheets/Fact-Sheet-Article-View/Article/476710/field-research-facility/

Lines 270/271: If these acronyms aren’t used again (LBT, LTT) they probably don’t need to be brought up here.

Line 311: was the nearshore wave transformation tool used to predict TWL or used to predict nearshore wave height/period/conditions?

Line 563: “runup models formulated elsewhere” isn’t entirely accurate since Holman was formulated elsewhere…

Line 715 – 717: Not sure I agree with this statement – It wasn’t beach slopes from Lidar data that outperformed the Satellite data – it was the slope definition. The performance of Lidar derived intertidal slopes looks/is explained (e.g., p^2 0.36, 0.38) similarly to coast satellite slopes when computing wave runup. To me, this isn’t a lidar versus satellite data issue – it’s the definition of the beach slope for extreme events issue.

# Editor report: Recommendation: Challenges and opportunities in predicting coastal total water levels from empirical runup models: Insights from major storms in Southeast Australia — R1/PR8

**DOI:** 10.1017/cft.2026.10043.pr8

**Affiliations:** Faculty of Engineering, UBC Institute for the Oceans and Fisheries, Canada

I have received the reviewer comments and recommendation on this manuscript. While the reviewer makes some very minor suggestions and comments, they have recommended that the manuscript be accepted for publication in Coastal Futures. I agree with their recommendation, and thank the authors for engaging with the previous review comments and improving the manuscript accordingly. The additional comments from the reviewer may be treated as suggestions that are small enough to be undertaken at the proofing stage. I encourage the authors to consider each of these, but will defer to their judgement on whether they make the suggested changes. 

Congratulations on the accepted manuscript, and I hope that you’ll consider Coastal Futures for the publication of similar work in the future.

# Editor report: Decision: Challenges and opportunities in predicting coastal total water levels from empirical runup models: Insights from major storms in Southeast Australia — R1/PR9

**DOI:** 10.1017/cft.2026.10043.pr9

**Affiliations:** Geography, Cambridge University, United Kingdom of Great Britain and Northern Ireland

No accompanying comment.

# Editor report: Recommendation: Challenges and opportunities in predicting coastal total water levels from empirical runup models: Insights from major storms in Southeast Australia — R2/PR11

**DOI:** 10.1017/cft.2026.10043.pr11

**Affiliations:** Faculty of Engineering, UBC Institute for the Oceans and Fisheries, Canada

My thanks to the authors for addressing the additional review comments on the revised manuscript. I’m happy to recommend that this is accepted for publication in Coastal Futures.

# Editor report: Decision: Challenges and opportunities in predicting coastal total water levels from empirical runup models: Insights from major storms in Southeast Australia — R2/PR12

**DOI:** 10.1017/cft.2026.10043.pr12

**Affiliations:** Geography, Cambridge University, United Kingdom of Great Britain and Northern Ireland

No accompanying comment.
